# Stretch Evolution of Electronic Coupling of the Thiophenyl Anchoring Group with Gold in Mechanically Controllable Break Junctions

**DOI:** 10.1021/acs.jpclett.3c00370

**Published:** 2023-06-15

**Authors:** Mani Lokamani, Filip Kilibarda, Florian Günther, Jeffrey Kelling, Alexander Strobel, Peter Zahn, Guido Juckeland, Kurt V. Gothelf, Elke Scheer, Sibylle Gemming, Artur Erbe

**Affiliations:** † Department of Information Services and Computing, Helmholtz-Zentrum Dresden-Rossendorf (HZDR), Bautzner Landstraße 400, 01328 Dresden, Germany; ‡ Institute of Ion Beam Physics and Materials Research, Helmholtz-Zentrum Dresden-Rossendorf (HZDR), Bautzner Landstraße 400, 01328 Dresden, Germany; ¶ Instituto de Física de São Carlos, Universidade de São Paulo, USP Av. Trabalhador saocarlense, 400, 13560-970, São Carlos, São Paulo, Brazil; § Institute of Physics, Technische Universität Chemnitz, 09107 Chemnitz, Germany; ∥ Department of Chemistry and Interdisciplinary Nanoscience Center, Centre for DNA Nanotechnology, iNANO, Gustav Wieds Vej 14, Aarhus C, 8000 Denmark; ⊥ Department of Physics, University of Konstanz, 78457 Konstanz, Germany

## Abstract

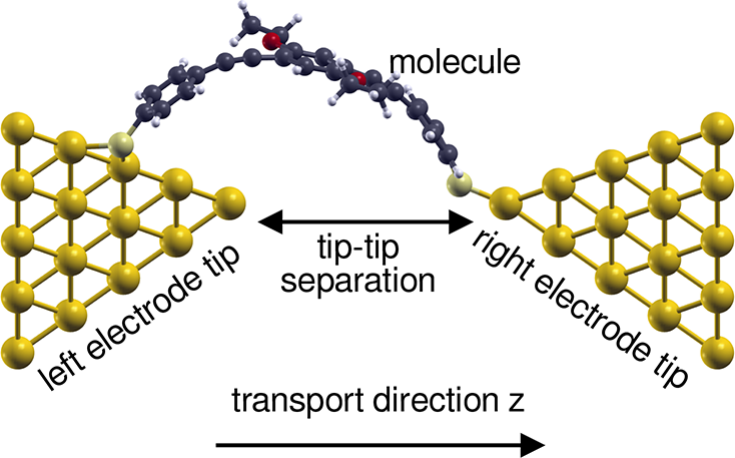

The current–voltage characteristics of a single-molecule junction are determined by the electronic coupling Γ between the electronic states of the electrodes and the dominant transport channel(s) of the molecule. Γ is profoundly affected by the choice of the anchoring groups and their binding positions on the tip facets and the tip–tip separation. In this work, mechanically controllable break junction experiments on the *N*,*N*′-bis(5-ethynylbenzenethiol-salicylidene)ethylenediamine are presented, in particular, the stretch evolution of Γ with increasing tip–tip separation. The stretch evolution of Γ is characterized by recurring local maxima and can be related to the deformation of the molecule and sliding of the anchoring groups above the tip facets and along the tip edges. A dynamic simulation approach is implemented to model the stretch evolution of Γ, which captures the experimentally observed features remarkably well and establishes a link to the microscopic structure of the single-molecule junction.

Many different extensions of the classical silicon technologies have been proposed to meet the constant demand of miniaturization of electronic devices during the past decades.^1−3^ Molecular electronics is one of the proposed extensions, which focuses on using single molecules as electronic components, which is the ultimate goal for miniaturization. Elementary electronic components like molecular transistors, switches, and rectifiers^4−8^ have already been demonstrated using single-molecule junctions. Recently, logic-in-memory operations in single-metallofullerene devices have been reported at room-temperature.^9^ Characterizing the physical and electronic properties of such single-molecular components requires selectivity and resolution at the nanoscale. This can be achieved using scanning tunneling microscopy (STM),^10,11^ atomic force microscopy (AFM),^12−14^ mechanically controllable break junctions (MCBJs),^15,16^ and electromigrated (EM) break junctions. The electron transport regimes, where these different measurement techniques are employed, have been discussed in detail in the literature.^17^ Among the above-mentioned techniques, MCBJs enable a more systematic study of the current–voltage (*I–V*) characteristics and conductance-breaking traces owing to the mechanical stability of the junctions. The stability of such molecule junctions is dictated by the binding strength of the anchoring group, deformation of the molecule, and thermal vibrations.^16,18−20^ Nevertheless, molecules can bind to the metallic electrodes at different positions and in various orientations depending on the specific chemical nature of the anchoring groups and the local symmetry of binding sites,^16,21−31^ which makes it a challenging task to elucidate the underlying physical mechanism governing the electronic behavior of single-molecule junctions.

Usually, 1-D conductance histograms and conductance-distance 2-D histograms are constructed from few hundreds or thousands of individual traces. The most probable *I–V* characteristic for a particular molecule–metal combination is associated with the most prominent conductance peak observed in the conductance histograms.^16,18,19,29,32,33^ The experimental characterization of single-molecule junctions via MCBJ is complemented with *ab initio* transport calculations, in order to estimate the relative positions of molecular levels, which act as transport channels, and the chemical potential of the metallic leads.^1,26,34−37^ However, the theoretical modeling of the broad distribution in the experimental conductance histograms is not feasible because (1) in MCBJ experiments, the adsorption geometries of the molecule between the metallic electrodes are *a priori* unknown,^38−40^ (2) many experimental factors remain elusive and are therefore not considered, and (3) the *ab initio* transport calculations are computationally expensive. Consequently, a limited number of energetically most favorable configurations are selected as representative junction geometries for theoretical investigations.^8,35,41,42^ However, recent technological advancements allow the simultaneous measurement of mechanical and electronic properties of single-molecule junctions, which enables deeper understanding of the structural information on junction geometries. For example, the most probable junction geometries for hexanedithiol and octanedithiol with gold electrodes in multiple traces can be probed in STM break junctions by applying a high-frequency sinusoidal mechanical signal during junction formation.^11^ In another study, metallocenes have been shown to form reproducible metal-molecule-metal junctions at low and room temperatures, that correlate with the atomic shape of the metal electrode and ineraction with electron-rich rings giving rise to extended conductance plateaus.^33^ Yet, to our knowledge, the evolution of junction geometries and associated electronic parameters of single-level model (SLM)^1^ has not been investigated in individual bridge openings so far.

In this work, we present a combined theoretical and experimental approach to describe the microstates of junction geometries and present the stretch evolution of the electronic coupling extracted from experimentally measured IV curves during individual bridge openings in a MCBJ setup for dithiolated-*N*,*N*′-bis(5-ethynylbenzenethiol-salicylidene)ethylenediamine cobalt complex (Co-Salen-S) (see Figure 1(a)).^43^ The evolution of the electronic coupling Γ with increasing tip–tip separation is accompanied by an initial decrease and a final increase, separated by a flat region of low Γ values with recurrent peaks. We use a novel high-throughput dynamic simulation approach for evaluating the stretch evolution of the SLM parameters by including a considerably large set of junction geometries and evaluating their respective nonequilibrium thermodynamical weights. Our theoretical approach is different from the previous works^8,35,42^ in that we consider multiple thermodynamically relevant configurations to evaluate the electronic properties of single-molecule junctions. We investigate the effects of the local symmetry of binding sites and of the deformation of the molecule upon anchoring on the *I–V* characteristics as the gap is widened and the anchoring groups either lock at specific binding sites or slide along the facets of the metallic electrodes toward the tip. The transmission functions *T*(*E*) from ballistic transport simulations for thermodynamically favorable junction geometries are used to extract the SLM parameters in the same way as for the measured IV curves, which allows a direct comparison with experimental findings. We relate the recurring peaks in the evolution of Γ with increasing tip–tip separation (*S*_tip–tip_) to the deformation of the molecule and steric boundary conditions at certain *S*_tip–tip_, which lead to energetically less favorable configurations. Finally, we present first efforts to establish a link between features observed in the evolution of the electronic coupling of stretching curves in MCBJs and the microstates of junction geometries, which are not accessible experimentally.

**Figure 1:**
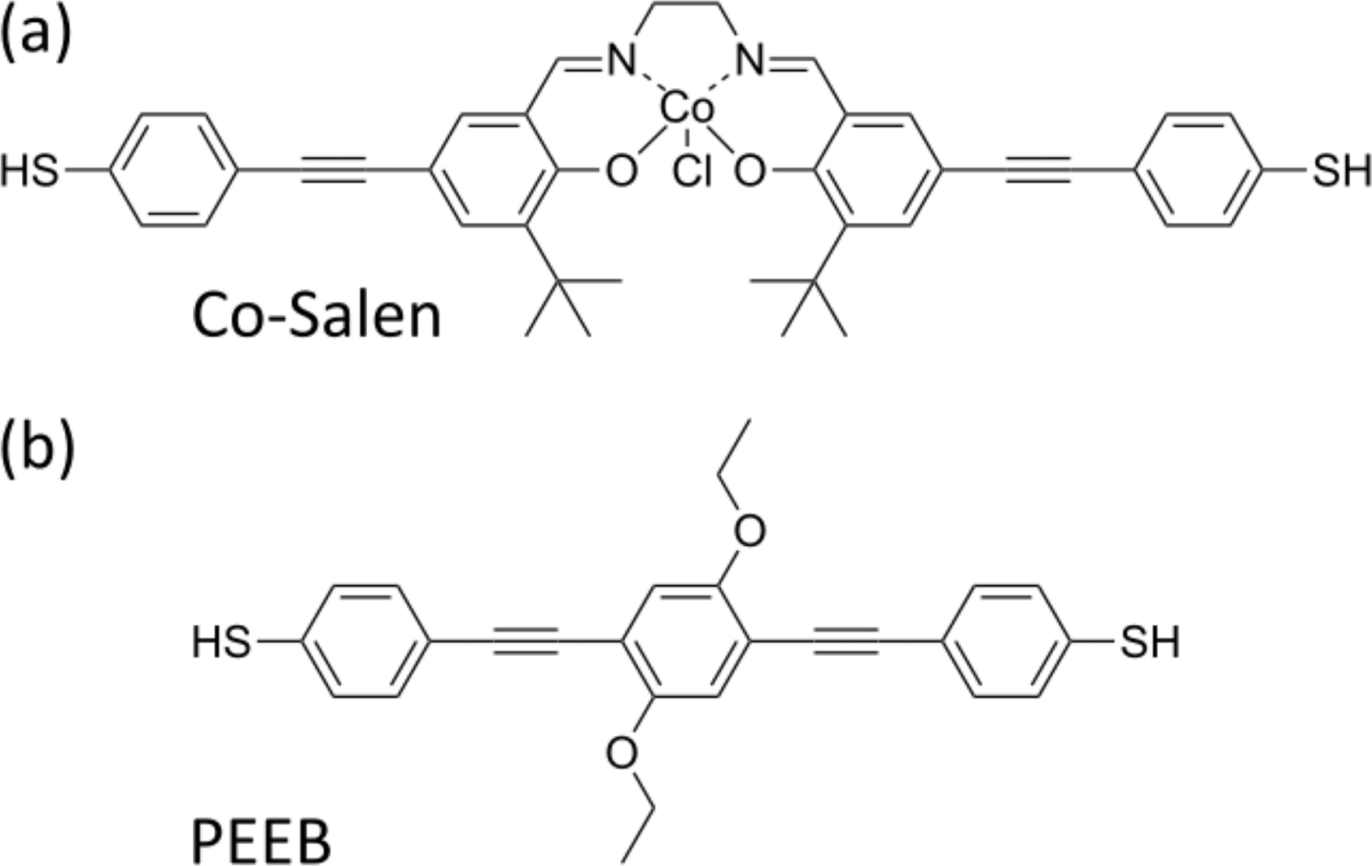
Schematic representation of (a) dithiolated-*N*,*N*′-bis(5-ethynylbenzenethiol-salicylidene)ethylenediamine cobalt complex (Co-Salen-S) and (b) dithiolated-1,4-bis(phenylethynyl)-2,5-bis(ethoxy)benzene (PEEB-S).

In an MCBJ setup, adjustable atomically sharp gold contacts^44,45^ are produced by deforming a lithographically manufactured nanoscopic gold constriction on a flexible substrate. The stretching of the nanoscopic gold constriction^46^ can be controlled to few Å/min. After breaking the direct metallic contact, this allows the formation of a single-molecule junction consisting of a single molecule connected to two metal electrodes in a solution containing the molecules to be measured. The experimental measurements were performed for the molecule Co-Salen-S (see Figure 1(a)). We chose thiol (SH) groups as anchoring groups since they provide stable bonds between molecules and metallic electrodes in charge transport measurements of single molecules. Moreover, sulfur atoms bind to high-symmetry top, hcp-, and fcc-hollow sites on the Au(111) surface (see SI Section S2) leading to a change of the *I–V* characteristics, which can be monitored in MCBJ experiments. The local binding environment of the SH linkers can be inferred from such measurements. For the MCBJ experiments, the acetyl-protected derivatives of Co-Salen-S were dissolved in toluene. A small amount of 20% ammonia solution in water was added as *in situ* deprotection agent right before the measurement. The solution was pipetted into the liquid cell on top of the lithographically defined nanogold contact of the MCBJ sample. After detecting the molecular signature^16^ in the electric signal obtained from the junction by a conductance measurement, the stretching of the junction is halted, and an *I–V* acquisition is initiated. The *I–V* measurements were recorded following a butterfly sweep (0 V → 0.8 V → – 0.8 V → 0.8 V → 0 V). Two full-range *I–V*-curves are obtained for a single bridge position. We wait at least 90 s between the *I–V* measurements following a butterfly sweep and until the standard deviation of the measured signal is lower than a threshold.^16^ The opening speed of the tips is of the order of 2 × 10^–12^ m/s. After completing the data acquisition, the stretching of the bridge is continued as long as the plateau persists and the *I–V* butterfly sweep is repeated. This procedure is performed until the junction is fully open, and no molecular signal is detected anymore. Before initiating another stretching, the junction is closed until the conductance value of 20 *G*_0_(47) is reached, where *G*_0_ denotes the quantum of conductance 7.748 × 10^–5^ S. All measurements were performed *in vivo* at room temperature. We performed 700 stretching cycles and rejected measurements that failed to fullfil the goodness-of-fit (GOF) criterion.^16^ We observed partial signatures of the stretch evolution of the electronic coupling (Γ^SE^) for Co-Salen-S in 5 measurements: 2 measurements for falling trend, 2 measurements for rising trend, and a single measurement for the intermiediate flattened region. We obtained only for a single measurement the complete Γ^SE^ with all the trends, namely the falling and rising trend, and an intermediate flattened region. For further details we refer to SI Sections S1 and S11.

We performed self-consistent density-functional-based tight-binding simulations for the geometry, electronic structure, and ballistic transport properties as implemented in the program package DFTB+ 20.1.^48^ The parameter set ”auorg-1-1” has been utilized in all calculations,^49^ which is an extension of the ”mio-1-1”^50^ parameter set. The ”mio-1-1” set has been developed for organic molecules including O, N, C, H, and S, whereas the ”auorg-1-1” set was designed to describe thiolates on gold nanoclusters.^51,52^ Dispersion corrections were included using the universal force field.^53^ The geometry relaxations were performed until the maximum force components were reduced to 0.0001 eV/Å. For calculating the transmission function, periodic boundary conditions in *x* and *y* directions and a shifted 2 × 2 Monkhorst scheme^54^ were used. In order to reduce the computational effort, we chose dithiolated-1,4-bis(phenylethynyl)-2,5-bis(ethoxy)benzene (PEEB-S) over Co-Salen-S, because Co-Salen-S binds to the electrodes via two peripheral PEEB-S moieties. Note that, it is not possible to determine the Γ^SE^ of PEEB-S in our MCBJ setup, because at shorter distances of the order of the dimensions of the PEEB-S, tunneling currents between the tip electrodes dominate over the molecular signature. Nonetheless, the influence of the central metal complex on the stretch evolution of Γ̅ can be neglected due to the length of the peripheral PEEB-S moieties. We evaluate the *I–V* characteristics by assuming that the electronic transport is dominated by a single-level model^1,36,37^ and by extracting ϵ_0_ and Γ, i.e., the energy and the coupling of that molecular orbital (see SI Sections S1 and S8). In addition to standard electronic structure methods such as density functional theory (DFT) or Hartree–Fock (HF), ballistic transport simulations are performed using the equilibrium Green’s function (EGF) formalism.^55−57^ Since the MCBJ experiments are performed in liquid conditions and the *I–V* measurements are recorded at the time scale of seconds, it can be assumed that the experimentally obtained SLM parameters do not originate from a single, energetically most favorable junction geometry, but rather represent a mean of many thermodynamically accessible configurations. Usually, the thermodynamically meaningful average is calculated by statistically averaging the quantities of interest for a selected set of snapshots from molecular dynamics trajectories.^58^ The main disadvantages of this approach are (a) an exhaustive sampling of the configuration space can only be achieved in long trajectories and (b) suitable exclusion algorithms for geometrically nearly identical snapshots are indispensible in order to avoid multiple evaluation of SLM parameters, using computationally expensive transport calculations.

Our dynamic simulation approach samples the configuration space systematically using a dense grid and addresses numerous thermodynamically accessible configurations. It is essential to include the thermodynamically accessible configurations in the case of thiol anchoring groups, because the local minima are separated from the global minimum through high barriers (see SI Section 2 on binding energy landscapes), which are unlikely to be surmounted during the short time scale of the experimental measurement. We employ a stochastic process (random walk) based on Metropolis transition probabilities,^59^ described by a master equation,^60^ and evaluate a transition matrix to propagate the probability density by utilizing the Metropolis criterion^59^ for neighboring configurations and zero elsewhere (see SI Section S3 for more details). Finally, we perform transport calculations for configurations which contribute most to the thermodynamical mean of the SLM parameters (see SI Sections S4 and S5). Our approach is computationally advantageous, because (a) the systematic sampling of the configuration space is trivially parallelized, and (b) the transport calculations are solely performed for thermodynamically relevant and unique geometrical configurations.

The experimentally measured stretch evolution of the electronic coupling (Γ^E^) and the theoretically determined stretch evolution of the electronic coupling () are shown in Figure 2. Please note the superscripts E and T to differentiate between the evolution of the electronic coupling determined experimentally and theoretically, resepectively.

**Figure 2:**
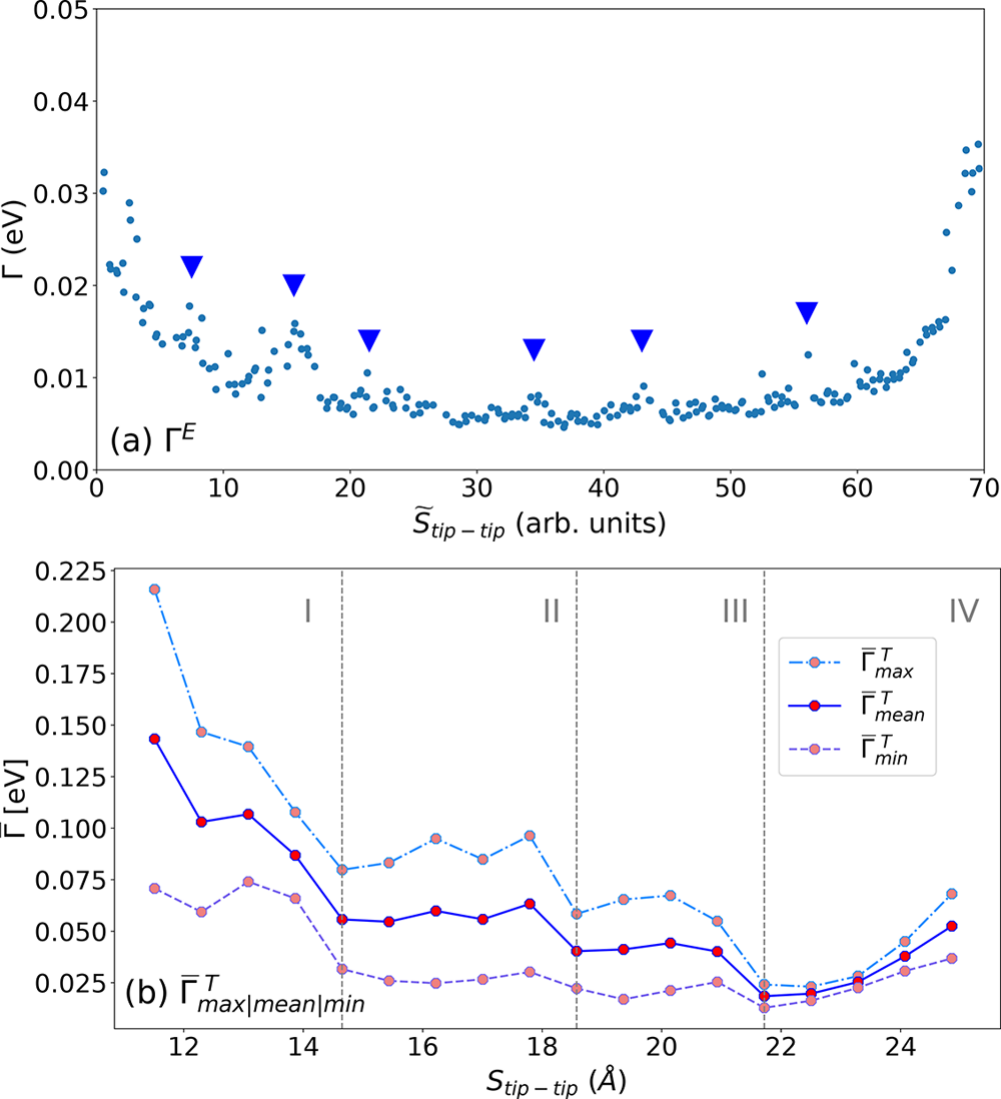
Experimentally measured stretch evolution of the electronic coupling (Γ^E^) for a single opening measurement of Co-Salen-S and theoretically determined stretch evolution of the electronic coupling () evaluated using the SLM applied to the transmission function *T*(*E*), which are calculated for 1000 thermodynamically most relevant configurations for individual tip–tip separation (*S*_tip–tip_). (a) Γ^E^ - the data points with peaks are marked using dark blue arrows. (b) , , and  reveal a falling trend for *S*_tip–tip_ interval (11.51 Å < *S*_tip–tip_ < 21.72 Å) and a rising trend for *S*_tip–tip_ > 21.72 Å. Peaks are visible for  at *S*_tip–tip_: 13.08, 17.79, and 21.72 Å, respectively. The four subdivisions I, II, III, and IV define the regions with the dominant anchoring position pairs: edge–edge, tip–edge, tip–edge + tip–tip, and tip–tip, respectively. Refer to the dominant anchoring positions in Figure 3(a).

The Γ^E^ values follow a general trend and reveal distinct peaks. In the initial opening phase, we notice a falling trend in Γ^E^ which flattens out for the larger part of the opening curve, only to be superseded by a surge in the final phase before break-off. The decrease in Γ^E^ is visible in the interval (0 < *S̃* < 20), the flattening out in the interval (20 < *S̃* < 50) and the increase thereafter (*S̃* > 50) until break-off. Please note that the tip–tip separation in experiments cannot be determined accurately and hence is denoted by *S̃*. Apart from this general trend in Γ^E^, we also observe peaks at various time intervals marked using dark blue arrows in Figure 2(a). Such peaks are also observed for other opening cycles (see SI section S1). Similarly,  reveals a drop in Γ^E^ for the *S*_tip–tip_ interval (11.51 Å < *S*_tip–tip_ < 21.72 Å) and a surge for *S*_tip–tip_ > 21.72 Å (see Figure 2(b)). Additionally, peaks are observed at *S*_tip–tip_ values of: 13.08, 17.79, and 21.72 Å.

In the following, we explore correlations between the stretch evolution of the electronic coupling and the stretch evolution of three geometrical descriptors: , , and . The geometrical descriptor  quantifies the fraction of configurations with dominating anchoring positions of the sulfur atoms on Au(111) facets on a scale between 0 and 1. The geometrical descriptors  and  quantify the mean curvature and the mean anchoring angle, respectively. The anchoring positions of the sulfur atoms on the Au(111) facets (AP) define the anchoring position doublet for both end groups on the left and right facets for each configuration. The mean curvature (mC) is a measure of the overall curvature of the molecular backbone (MB) of PEEB-S. The anchoring angle (AA) specifies the angle measured between the S–C-bond and the facet normal. The geometrical descriptors collectively capture both the deformation of the molecule and the anchoring position of the sulfur atoms at the gold surfaces. For more details on AP, mC, and AA, we refer to the SI Sections S5, S6, and S7, respectively. The theoretically determined stretch evolution of these geometrical descriptors is then averaged using the same statistical approach employed for the SLM parameters (see SI Section S3). The theoretically determined *stretch-evolution* of AP, mC, and AA are defined as , , and  and shown in Figure 3. The trend associated with  and its peaks can be explained by analyzing , , and .

**Figure 3:**
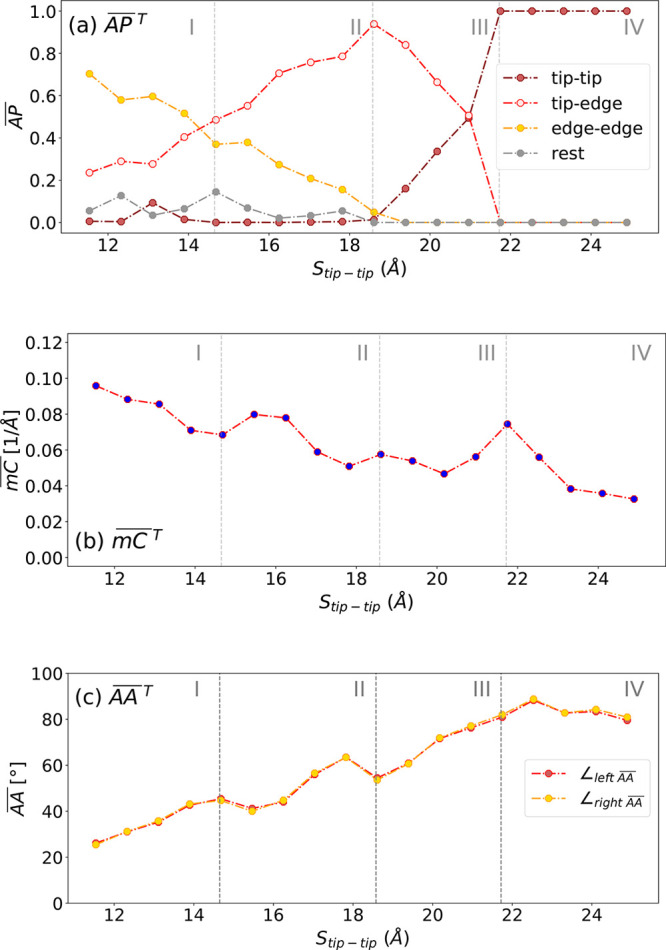
(a) Theoretically determined stretch evolution of the mean anchoring position on the Au(111) facets () - four distinct *S*_tip–tip_ regions are discernible. For *S*_tip–tip_ < 13.87 Å, the dominating mean anchoring positions of the sulfur atoms on the Au(111) facets () are either edge–edge or tip–edge. For *S*_tip–tip_ between 14.65 and 18.58 Å, the tip-edge  dominates over edge–edge . The contribution of edge–edge  diminishes for *S*_tip–tip_ > 19.36 Å with the emergence of tip–tip . For *S*_tip–tip_ between 19.36 and 20.9 Å, the tip-edge  dominate. For *S*_tip–tip_ > 21.72 Å, the  are solely tip–tip. (b) Theoretically determined stretch evolution of the mean curvature () - an overall falling trend is discernible. Trough-like features are visible at *S*_tip–tip_: 13.87, 17.79, and 20.9 Å, respectively. A distinct sharp peak is visible at *S*_tip–tip_: 21.72 Å. (c) Theoretically determined stretch evolution of the mean anchoring angle () - An overall rising trend until saturation is discernible. Peaks are visible at *S*_tip–tip_: 15.44 and 18.58 Å.

We recall that the stretch evolution of the SLM parameters can be determined theoretically from the transmission function (see SI Sections S4 and S8). Ballistic transport calculations were performed for the 1000 thermodynamically most relevant configurations (see SI Sections S2, S3, S4, and S5).

 as a function of the tip–tip separation (Figure 3(a)) reveals four distinct regions, (I) *S*_tip–tip_ < 13.9 Å, (II) 13.9 Å < *S*_tip–tip_ < 14.7 Å, (III) 14.7 Å < *S*_tip–tip_ < 20.9 Å, and (IV) *S*_tip–tip_ > 20.9 Å. At short *S*_tip–tip_ (region I) and large *S*_tip–tip_ (region IV) the dominant anchoring positions are edge–edge and tip–tip, respectively. In region I, the dominant electronic channel can interact with additional surface states from the adjacent facets which may lead to an enhanced electronic coupling Γ. In region IV, the molecule is anchored symmetrically at the tip apexes assuming a planar configuration and can form an optimal anchoring angle with the facet normal vectors (∠_LA_ - left anchoring angle, ∠_RA_ - right anchoring angle) (see SI Section S7) that enhances the electronic coupling Γ significantly.

In intermediate regions II and III, mixed tip-edge configurations become dominant, which lowers the electronic coupling Γ in comparison to the symmetric cases edge–edge and tip–tip. In particular, the planar conformations in these regions result in trough-like features of the  curve (see Figure 3(b)) at *S*_tip–tip_ values of 13.08, 17.79, and 21.72 Å, coinciding with the peaks in the theoretically determined stretch evolution of  () at *S*_tip–tip_: 13.08 Å, and of the theoretically determined stretch evolution of  () at *S*_tip–tip_: 17.79 and 21.72 Å, respectively. Furthermore, the minima of the curvature coincide also with peaks in the evolution of the , implying that the electronic coupling rises at certain distances *S*_tip–tip_, where the molecule is predominantly planar. Similarly, the  adopts peaks especially at *S*_tip–tip_: 15.44 and 18.58 Å, which may also increase the electronic coupling further. For *S*_tip–tip_ > 22.5 Å the dominant  are tip–tip. When anchored on both ends at the tip regions, the PEEB-S molecule can assume optimal anchoring angles due to additional rotational degrees of freedom; there it can rotate freely about the molecular backbone. The  saturates for *S*_tip–tip_ > 20.15 Å to values between 70° and 90°, suggesting that most of the relevant configurations have formed optimal anchoring angles and form a fully planar PEEB-S molecule aligned in transport direction. Selected configurations are shown in SI Section S12.

The effect of the bending plays a more dominant role than the effect of forming the optimal anchoring angles. This is further confirmed by the sharp dip of the  curve at *S*_tip–tip_ = 18.58 Å followed by a surge up to a  value of 0.06 eV until break-off in theoretically determined stretch evolution of Γ̅ () (Compared with the steep increase at *S*_tip–tip_ = 18.58 Å followed by a drop in ). A cross-correlation plot between *S*_tip–tip_, , , , , , and  is shown in SI Section S10 for completeness.

In addition, the electronic coupling of the site-specific anchoring of the molecule on the Au(111) facets can be visualized using the real-space projection of the stretch evolution of the electronic coupling. The real-space projection of Γ of a single configuration is obtained by replacing the clamped molecule between Au(111) electrodes with a pair of colored circles at the anchoring sulfur atoms, where the color and the radius of the circles represent the Γ_mean_ (see Figure 4(c) and SI Section S9).

**Figure 4:**
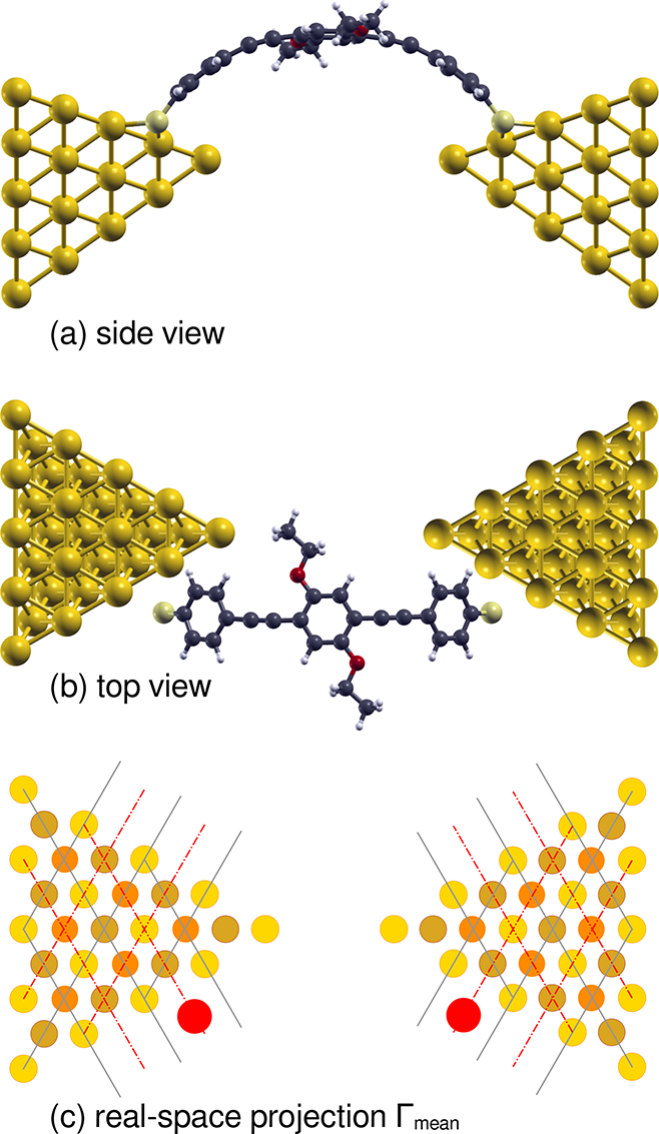
Real-space projection of Γ_mean_ (a) side view and (b) top view (not to scale) of the configuration with strongest coupling for the *S*_tip–tip_ = 12.33 Å. (c) The value of Γ_mean_ is represented by red circles at the sites of the anchoring sulfur atoms on the left and right electrodes in top view. The gold atoms of the electrodes are colored to depict the top, hcp-hollow, and fcc-hollow sites using yellow, brown, and salmon colored circles, respectively. The solid gray and dotted red lines as shown to indicate the anchoring of the sulfur atoms at top and bridge sites along the Au(111) facet edges of both the left and right electrodes.

In Figure 5, the real-space projection of the theoretically determined Γ_mean_ is shown for the 50 configurations with the highest random walk weights for each *S*_tip–tip_. We analyze three intervals for the tip–tip separation: (1) 13.9 Å < *S*_tip–tip_ < 14.7 Å, (2) 14.7 Å < *S*_tip–tip_ < 20.9 Å, and (3) *S*_tip–tip_ > 20.9 Å separately.

**Figure 5:**
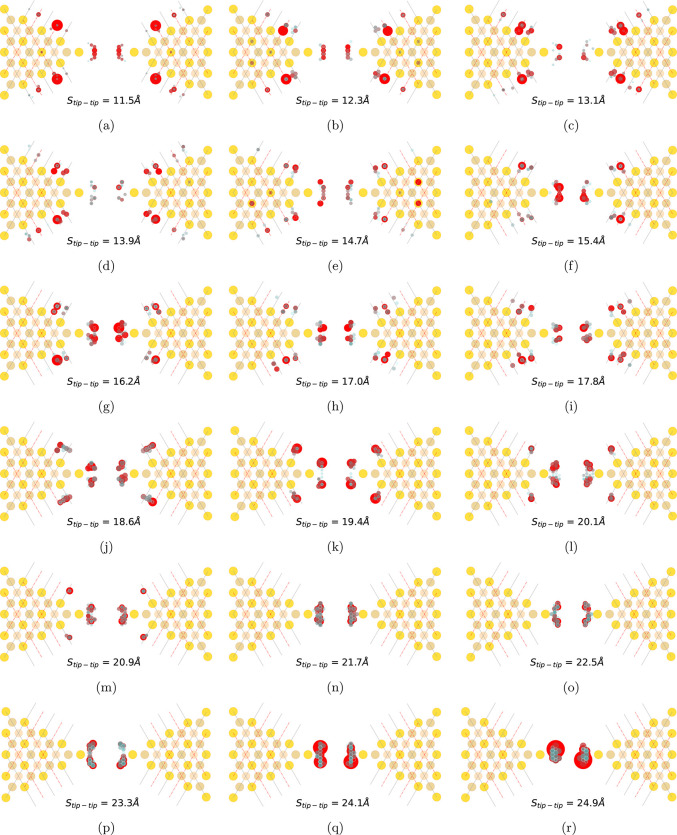
Real-space projection of the theoretically determined stretch evolution of Γ_mean_ for those 50 configurations for each *S*_tip–tip_, with the highest random walk weights. The gold electrodes are represented using yellow, brown, and salmon colored circles, representing the top, hcp-hollow, and fcc-hollow sites, respectively. The other colored circles appear in pairs, which reveal the binding sites for the anchoring sulfur atoms on the left and the right gold electrode. Higher and lower Γ_mean_ values are represented using a color scale spanning from red to gray, augmented together with the size of circle. Additionally, solid red and dotted gray lines are placed to differentiate between bridge sites and top sites along the Au(111) facet edges, respectively.

In the first interval (see Figure 5(a)–(e)), the anchoring positions with the strongest electronic coupling shift from bridge to top sites along the Au(111) facet edges. The larger red circles situated at bridge sites for *S*_tip–tip_ 11.5 and 12.3 Å evolve into smaller red circles closer to the top sites for *S*_tip–tip_ 13.1, 13.9, and 14.7 Å. For the corresponding *S*_tip–tip_ interval  reveals a falling trend, suggesting that at bridge sites the transport channel couples more strongly to the metal states than at the top sites (Figure 2(b)).

Using similar arguments, we dintinguish two trends in the second interval. The first trend (see Figure 5 (e) → (f) → (g) → (h) → (i)) reveals a shift in the dominating anchoring positions from top sites toward bridge sites along the Au(111) facet edges. For the corresponding *S*_tip–tip_ interval in Figure 2(b), we notice a rising trend in  and the maxima at *S*_tip–tip_ values of 16.2 and 17.8 Å. The second trend (see Figure 5 (j) → (k) → (l) → (m) → (n)) reveals a shift in the dominating anchoring positions from bridge sites toward top sites along the Au(111) facet edges. For the corresponding *S*_tip–tip_ interval in Figure 2(b), the  reveals a falling trend to the global minimum at *S*_tip–tip_ (21.7 Å), where the dominating anchoring positions are centered around the top sites. These trends coincide with the earlier observation that the metallic state at the bridge site couples more strongly to the molecular transport channel than that at the top site.

In the third interval for *S*_tip–tip_ > 21.7 Å, the molecule predominantely anchors at the tip regions of both electrodes (see Figure 5(n)–(o)), adopts planar geometry (see Figure 3(b)) and optimal anchoring angles (see Figure 3(c)). This leads to the enhancement in the electronic coupling, which is observed as a surge in  (see Figure 2(b)).

In summary, the trend observed in the stretch evolution of Γ_mean_ originates from the complex interplay of the anchoring sulfur atoms sliding between bridge and top sites along the Au(111) facet edges, the molecule ideally adopting a planar geometry and the optimal anchoring angle at the contact. At small *S*_tip–tip_, the anchoring positions with the strongest electronic coupling are centered around the bridge sites along the Au(111) facet edges. At large *S*_tip–tip_, the molecule anchors to the apex region of the tips, where it experiences less steric constraints and can more readily assume an optimal geometry and anchoring angle. This is reflected in the increase of  before break-off. In the intermediate interval, mixed tip-edge configurations become dominant, which exhibit lower values for the overall transmission than the symmetric edge–edge and tip–tip configurations. Peaks and minima alternate in this region as the molecule slides over bridge and top positions along the edge.

To conclude, we present a combined theoretical and experimental approach to describe the microstates of junction geometries in individual bridge opening curves in MCBJs. We employ a novel, high-throughput dynamic simulation approach to model the theoretically determined stretch evolution of the electronic coupling Γ. We perform transport calculations using the self-consistent density-functional-based tight-binding approach and the Green’s function formalism for not only just one representative but many thermodynamically relevant configurations. We average the obtained single-level model parameters (ϵ_0_ and Γ) using statistical weights obtained from random walk Metropolis simulations. The behavior of the theoretically determined stretch evolution of the electronic coupling () reflects the experimentally measured stretch evolution of the electronic coupling (Γ^E^) well. We associate the recurring maxima of Γ with an overall reduction of curvature of the molecular backbone, increased anchoring angle, and symmetric, preferentially edge–edge or tip–tip configurations.

Our theoretical approach in combination with MCBJ experiments elucidates the dependency of the electronic coupling of thiol anchoring groups on the distance of the gold contacts, which has so far not been reported in the literature. We correlate strong electronic coupling at short and large *S*_tip–tip_ and weak electronic coupling in the intermediate region to geometrical descriptors: mean curvature, mean anchoring positions of the sulfur atoms on the Au(111) facets, and mean anchoring angle. The comparison of our theoretical analysis with the experimental measurements suggests that a strong electronic coupling is obtained for *S*_tip–tip_, where symmetrical contact geometries are statistically more common. The peaks in the intermediate region in the evolution of Γ arise from the contribution of statistically dominant, energetically less favorable molecule-tip-Au(111) structures, which are asymmetric due to steric boundary conditions. Thus, with our novel approach, we establish a link between the evolution of Γ of stretching curves in MCBJ measurements and the microscopic structure of a single molecule anchored between gold electrodes.

## Supporting Information Available

Additional details on experimental setup and on theoretical methods (PDF)
Transparent Peer Review report available (PDF)
