# Supplementary material for: Stretch Evolution of Electronic Coupling of the Thiophenyl Anchoring Group with Gold in Mechanically Controllable Break Junctions
Source: J Phys Chem Lett. 2023 Jun 15;14(24):5709–17. doi: 10.1021/acs.jpclett.3c00370 (PMC10291638; doi:10.1021/acs.jpclett.3c00370)
Supplement: Supplementary file 1 — jz3c00370_si_001.pdf [file jz3c00370_si_001.pdf]

Supplementary Information: Stretch Evolution of Electronic Coupling of the Thiophenyl Anchoring Group with Gold in Mechanically Controllable Break Junctions

Mani Lokamani,[*,†] Filip Kilibarda,[‡] Florian Günther,[¶] Jeffrey Kelling,[†] Alexander Strobel,[‡] Peter Zahn,[‡] Guido Juckeland,[†] Kurt V. Gothelf,[§] Elke Scheer,[‖] Sibylle Gemming,[‡,⊥] and Artur Erbe[‡]

†*Department of Information Services and Computing, Helmholtz-Zentrum Dresden-Rossendorf (HZDR), Bautzner Landstraße 400, 01328 Dresden, Germany*

‡*Institute of Ion Beam Physics and Materials Research, Helmholtz-Zentrum Dresden-Rossendorf (HZDR), Bautzner Landstraße 400, 01328 Dresden, Germany*

¶*Instituto de Física de São Carlos, Universidade de São Paulo - USP Av. Trabalhador saocarlense, 400, CP. 369 - CEP 13560-970, São Carlos - SP, Brazil*

§*Department of Chemistry and Interdisciplinary Nanoscience Center, Centre for DNA Nanotechnology, iNANO, Gustav Wieds Vej 14, Aarhus C, 8000 Denmark*

‖*Department of Physics, University of Konstanz, 78457 Konstanz, Germany*

⊥*Institute of Physics, Technische Universität Chemnitz, 09107, Chemnitz, Germany*

E-mail: m.lokamani@hzdr.de

S1: Opening Curves

The experimentally measured stretch evolution of the SLM parameters is shown for 2 different opening cycles in Figure S1. In Figures S1(a)-(b), the data points for ϵ_0^E oscillate about the mean values of 0.74 eV and 0.78 eV, respectively. In contrast, the Γ^E reveals a general trend and distinct peak-like features. In Figure S1(c), the Γ^E reveals a rising trend. In Figure S1(d), a falling trend is visible. Distinct peak-like features are visible for the Γ^E in both opening cycles.

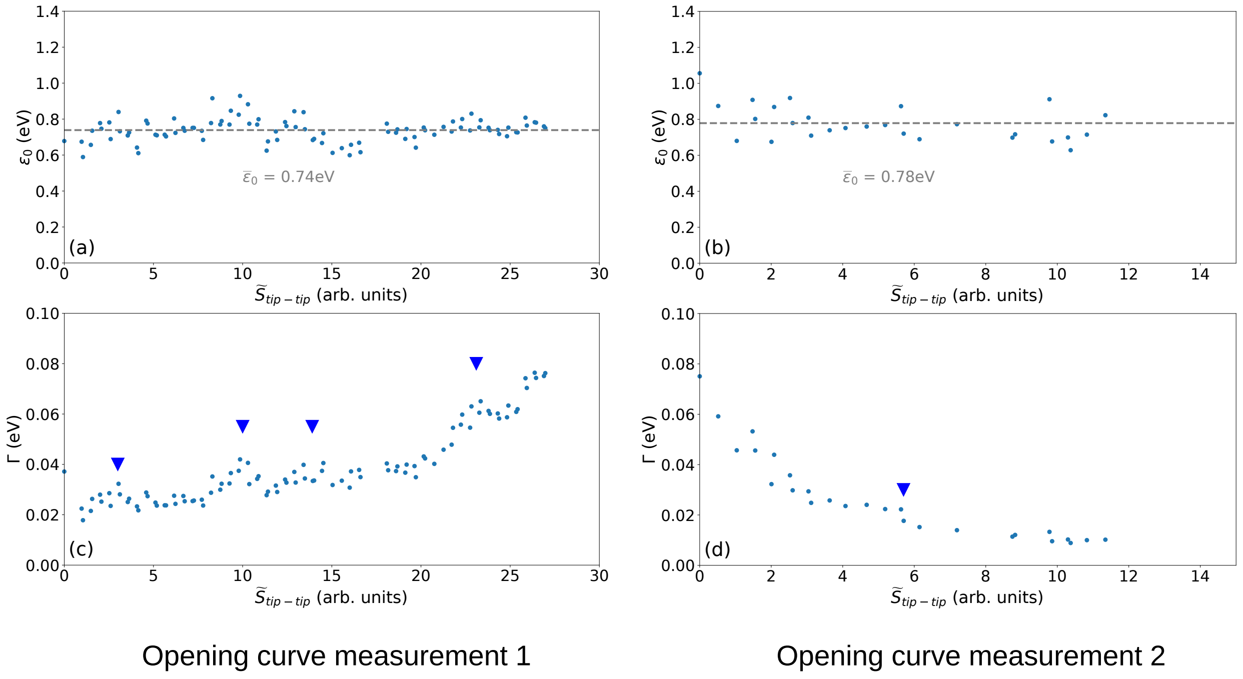

Figure S1: Experimentally measured stretch evolution of the SLM parameters $(\epsilon_0^E|\Gamma^E)$ for 2 different opening cycles. The data points for ϵ_0^E oscillate about the mean values, not revealing any particular trend or features. In contrast, the Γ^E reveal falling/rising trends and plateaus with distinct peak-like features.

Data Evaluation

To determine the stretch evolution of Γ, the recorded current-voltage $(I - V)$ curves were fitted using the single-level model (SLM). The main premise of this approach is that the $I-V$ characteristics and consequently the $T(E)$ of the molecule junction can be characterized by ϵ_0, Γ_{max} and Γ_{min}. ϵ_0 is the energy level of the molecular orbital closest to the Fermi level (E_F) of the metallic leads, which acts as the dominating transport channel. The coupling strengths of the dominant molecular orbital to the electronic states of the contacts are denoted by Γ_{max} and Γ_{min}. In case the molecular orbital closest to E_F is the highest-occupied molecular orbital (HOMO) or the lowest-unoccupied molecular orbital (LUMO), the electronic transport is hole or electron dominated, respectively.

The transmission as a function of energy E is given as:

$$T(E) = \frac{4\Gamma_{max}\Gamma_{min}}{(E - \epsilon_0)^2 + (\Gamma_{max} + \Gamma_{min})^2} \tag{1}$$

Note, if the coupling strengths of the dominant molecular orbital to the electronic states of the contacts are of the same order, i.e. $\Gamma_{max} \approx \Gamma_{min}$, the transmission as a function of energy E assumes a form[1] given by:

$$T(E) = \frac{4\Gamma^2}{(E - \epsilon_0)^2 + 4\Gamma^2} \tag{2}$$

Note, some of the measurements give values for ϵ_0, Γ_{max} and Γ_{min}, that differ from the average values by orders of magnitude. The origin of these large deviations is unclear, as they may be related to junctions at very small distances, in which the contribution by direct tunneling becomes dominant. In addition, a large number of measurements results in $I - V$ curves, which significantly deviate from the predictions made by the SLM. These measurements may be associated to the formation of clusters of molecules or the presence of more than one transport channel in a single molecule. In this study, such measurements are rejected by means of a goodness-of-fit (GOF) criterion with adjusted R^2. We select curves with GOF > 0.99 ,[2] that fit perfectly to the SLM (see Figure S2).

Although in mechanically controllable break junction (MCBJ) experiments the tip opening can be controlled at the sub-nanometer scale, the exact tip-tip separation at any given moment is hard to quantify precisely. An opening cycle is initiated by short-circuiting the tips until at least $20\,\mathrm{G}_0$ conductance is attained, where G_0 denotes the quantum of conductance $7.748 \times 10^{-5}\,\mathrm{S}$. This procedure may change the shape of the tips drastically, such that the experimental tip-tip separation is bound to vary in different opening measurements for the same position of the pushing rod. In addition, the nonlinear behavior of the substrate flexure[3] can also affect the envisaged linear change in the tip-tip separation with the pushing rod.[2] However, once the tip formation has concluded, the tips retain their shape during the opening cycle before break-up, unless the tractive force fails to induce the stretching of the molecule or the sliding of the anchor groups on the tip facets. The raw data that is recorded during the experiment is therefore the time and the position of the motor that controls the pushing rod and cannot be mapped to an exact tip-tip separation. Therefore, the experimental tip-tip separation are given in units of d_0, which is assumed to be constant for each individual opening cycle. d_0 denotes the position of the motor used for the pushing rod.

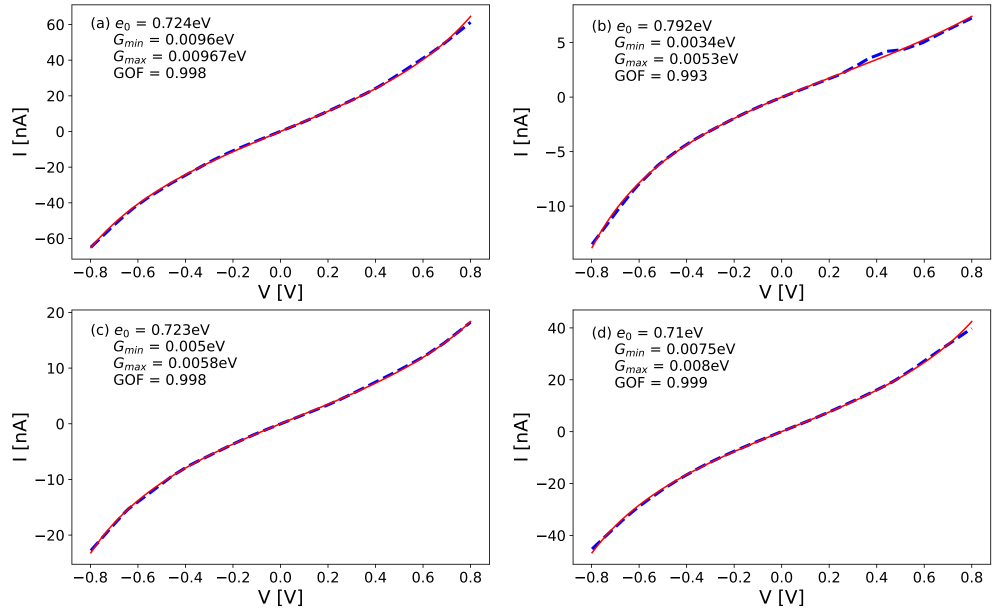

Figure S2: Four examples of IV-characteristics and their fits. The dashed blue curves are experimental measurements and the pink solid curves are the corresponding fits. The values of the single-level model parameters (labeled as ϵ_0, Γ_{min} and Γ_{max}) and the goodness-of-fit are given for all the fits.

S2: Binding Energy Landscape: Thiophenyl on Au(111) Facet

The binding energy landscape for thiophenyl on Au(111)-facet is determined for three constraint relaxation schemes as shown in Figure S3. The schemes differ in preservation of the aligment of the main axis during the relaxation simulation. The main axis of the dithiolated-1,4-bis(phenylethynyl)-2,5-bis(ethoxy)benzene (PEEB-S) molecule defined using the nearest and the farthest carbon atoms to the anchoring sulfur atom and the sulfur atom itself. The schemes shown in Figure S3(a) and (b), describes the binding energy landscape, where the main axis forms an angle of 109.5° and 19.5° to the Au(111)-facet, respectively. In the scheme shown in Figure S3(c), the main axis can adopt any angle to the Au(111)-facet. The differences between the highest/lowest binding energies in the energy landscapes associated with the schemes $LS_1/LS_2/LS_3$ amounts to 1.21 eV/1.88 eV/1.58 eV, respectively. The energetically most/least favorable configurations for the binding of PEEB-S to the Au(111)-facet are obtained for constraint relaxation schemes 3/2, respectively. The energy difference between the energetically most ($E_{min}LS_3$) and least ($E_{max}LS_2$) favorable configuration amounts to 2.0 eV.

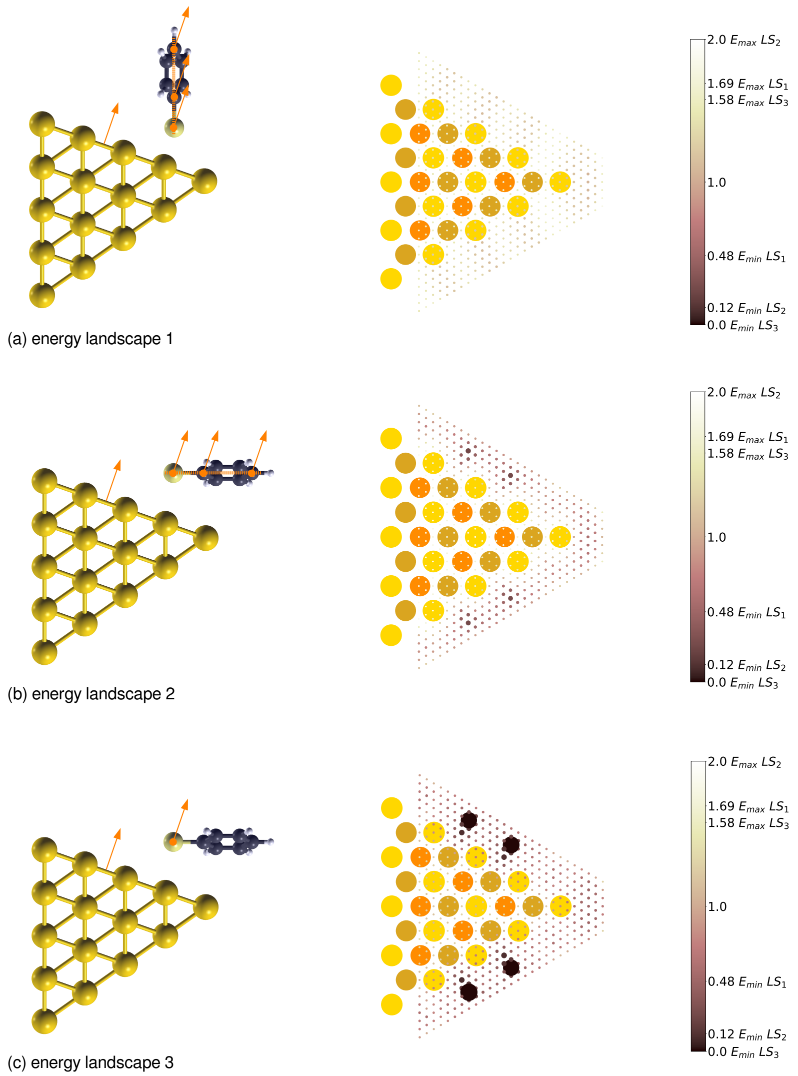

Figure S3: The binding energy landscape of thiophenyl on Au(111)-facets for three different constraint relaxation schemes. a) vertical constraint - the main axis forms an angle of 90° to the xy-plane. b) horizontal constraint - the main axis is lies in the xy-plane. c) free relaxation- the molecule as allowed to move freely. The main axis of the PEEB-S molecule is composed of the nearest and farthest carbon atom to the anchoring sulfur atom and the sulfur atom itself. In all three scenarios, the anchoring sulfur atom is allowed to move along the facet normal vector ($\vec{n}_{facet}$) (shown using orange arrows). The relative binding energies of adsorbed thiophenyl at each anchoring position are shown in the top view directed along the $\vec{n}_{facet}$ using dark-red circles of varying sizes and color saturation. Darker and larger circles correspond to energetically more favorable anchoring positions. The high-symmetry top, hcp-, and fcc-hollow sites are shown using yellow, dark yellow and dark orange colored circles, respectively.

S3: Comparison of Boltzmann and Random-Walk Weights

A comparison of Boltzmann and random-walk weights is shown for 161 889 anchored configurations at tip-tip separation ($S_{tip-tip}$) = 11.54 Å using red and blue data points in Figure S4(a)-(b), respectively. The relative energies (gray data points) are plotted in ascending order. Considering the relative short timescale of the experimental measurements, a Boltzmann state distribution is far from being the optimal choice. In the case of thiol-groups, the local minima are separated from the global minimum through high barriers, that are unlikely to be surmounted during the short timescale of the experimental measurement. In order to evaluate the (non-)equilibrium weights, we use a stochastic process (random-walk) based on Metropolis transition probabilities,[4] described by a master equation.[5] We evaluate a transition matrix to propagate the probability density by utilizing the Metropolis criteria[4] for neighboring configurations and zero elsewhere. Neighboring configurations differ only in one of the anchoring positions by a single grid spacing.

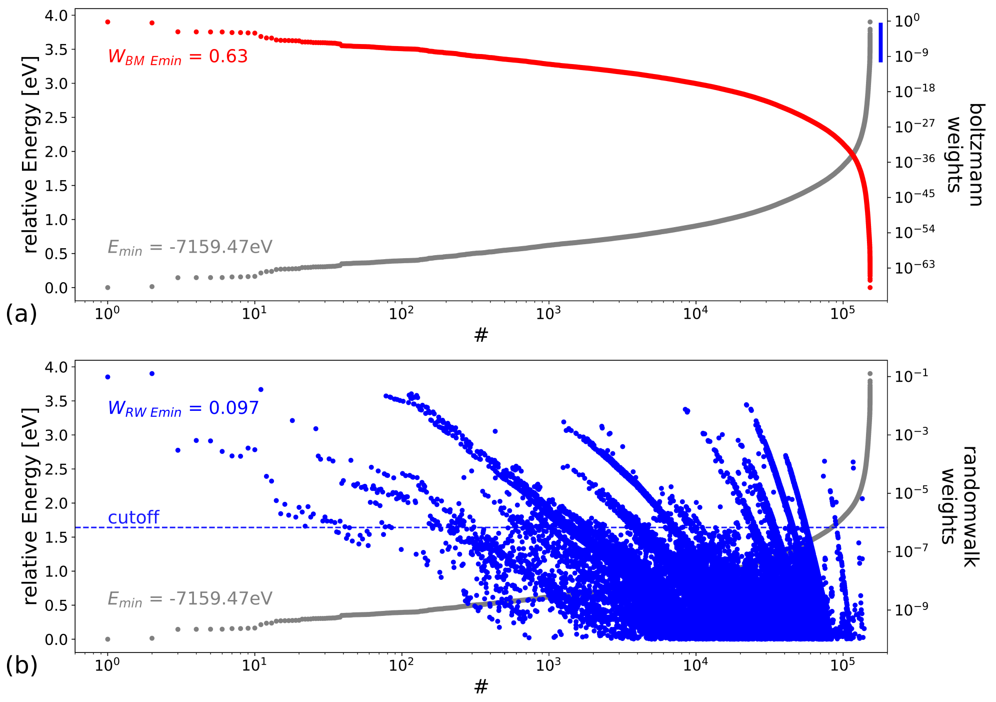

Figure S4: Comparison of Boltzmann and random-walk weights for anchored configurations at $S_{tip-tip}$ = 11.54 Å. (a) The total energies (gray data points) of the 161 889 configuration considered are plotted in increasing order. The Boltzmann weights (red data points) mirror the red curve vertically as expected. The Boltzmann weight of the energetically most favorable configuration amounts to 0.63 . (b) The corresponding random-walk weights for the 161 889 configuration are shown using blue data points. The random-walk weight of the thermodynamically most favorable configuration amounts to 0.097 . The cutoff (dashed line) separates the 1000 configurations with leading random-walk weights. The blue solid line in (a) exemplifies the difference in scales between the Boltzmann and random-walk weights.

The comparison of random-walk weights above the cutoff line in Figure S4(b) with the corresponding Boltzmann weights in Figure S4(a), foreshadows the importance of considering configurations, which at first glance seem irrelevant on grounds of inferior energetics. The cutoff marks 1000 thermodynamically most favorable configurations. A closer look at the *step-like* increments (see Figure S5(a)) in the cummulative sums of (non-)equilibrium weights exemplifies the following: (1) energetically less favorable configurations with small/negligible Boltzmann weights are associated with significant random-walk weights and, (2) energetically less favorable configurations can be thermodynamically relevant and can contribute considerably to the statistical evaluation of single-level model parameters (ϵ_0 and Γ).

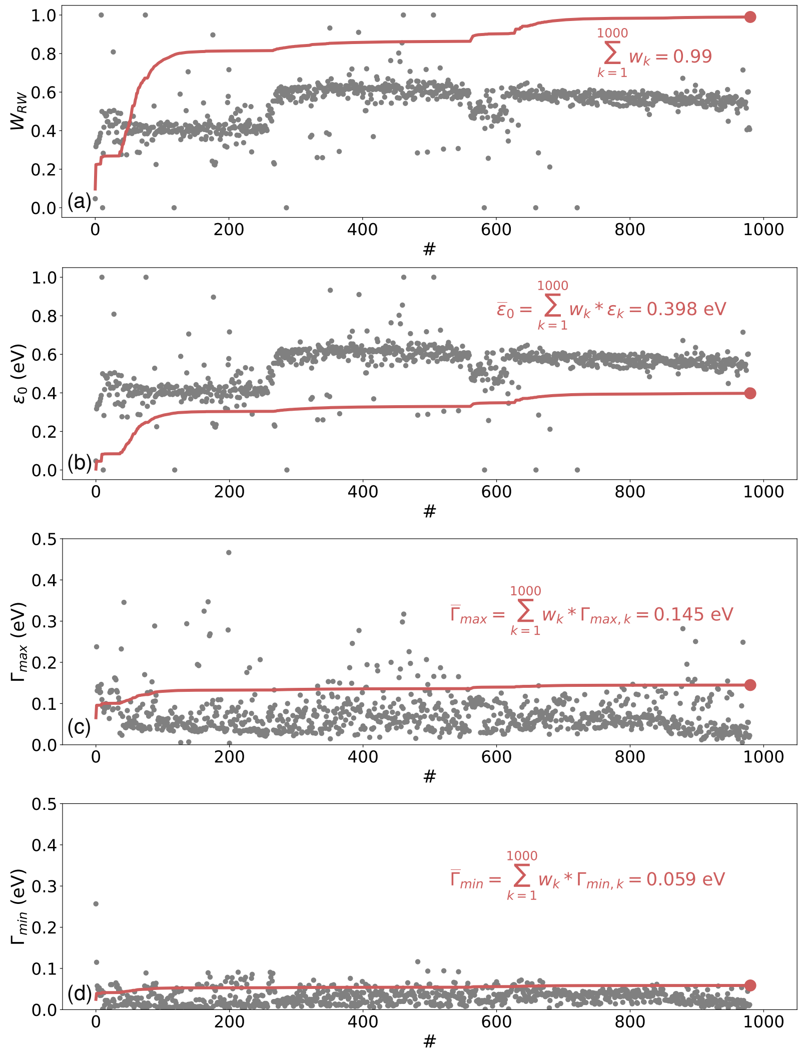

Figure S5: (a) Cumulative sum of random-walk weights. (b)-(d) Statistically averaged single-level model parameters (ϵ_0 and Γ) of ϵ_0, Γ_{max} and Γ_{min} , respectively, for $S_{tip-tip}$ = 11.54 Å. The *step-like* increments in (a)-(c) suggest, that the energetically less favorable configurations with small/negligible Boltzmann weights have significant random-walk weights and non-negligible contribution towards the statistical evaluation of ϵ_0 and Γ.

S4: Simulation setup

The transport setup consists of an extendend molecule which is composed of a central scattering region and a buffer region on each side, and semi-infinite leads in the direction of transport (see Figure S6). The scattering region consists of tetrahedron-shaped tips with a base length of 4×2.855 Å and the PEEB-S molecule attached to both facets through sulfur endgroups. The buffer layers and the semi-infinite leads have a base length of 5×2.855 Å in the xy-plane and contain 3 gold layers with face-centered-cubic local-coordination environment. The whole setup is periodic transversal to the z-direction of electronic transport. The left and right facets of the tetrahedron-shaped tips as shown in Figure S7(a), are inclined at an angle of 19.5° relative to the transport direction z.

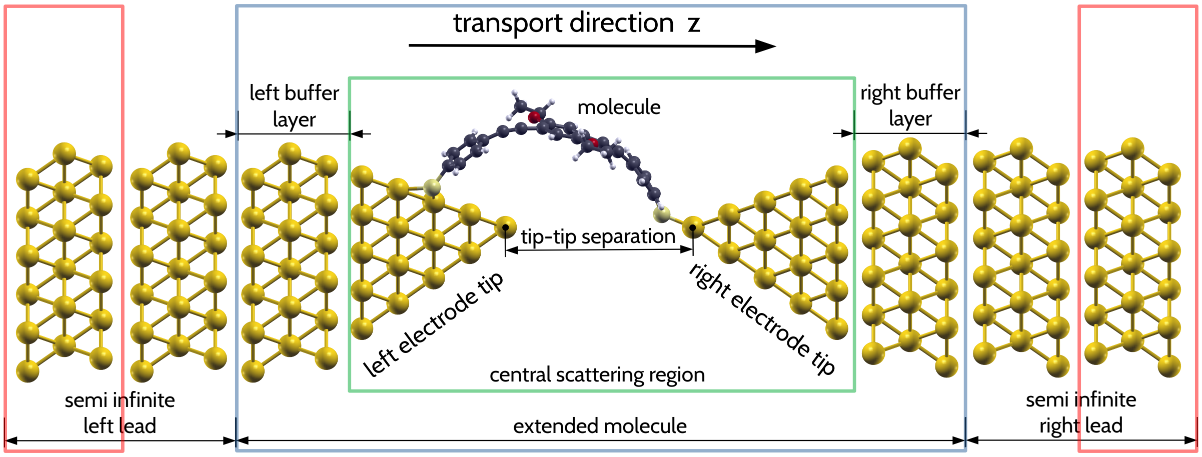

Figure S6: The transport setup consists of an extendend molecule (blue frame) which is composed of a central scattering region and a buffer region on each side, and semi-infinite left/right electrodes (red frames). The central scattering region (green frame) consists of left/right electrode tips and the clamped PEEB-S molecule. The buffer-layers are composed of three gold layers of size 5×2.855 Å with face-centered-cubic local-coordination environment (fcc-LCE). The electrode tips are tetrahedron-shaped with edge-length of 4×2.855 Å conserving the fcc-LCE of the facets. The semi-infinite left and right electrodes are formed by repeating three fcc-layers of gold as the buffer layers.

S5: High-throughput simulations

During the time scales of the measurements, the molecule may assume various configurations between the electrodes. For instance, the thiol groups can diffuse along the facets or get trapped in local minima in the energy landscape (see SI Section S2 for details on energy landscape for thiol anchoring groups). Concurrently, the molecule prefers to form the optimally directed Au-S anchoring angle.[6] Together, these processes can lead to a geometric deformation in form of streching and bending of the molecular backbone (MB) of PEEB-S molecule. Both, the anchoring positions of the sulfur atoms on the tip-facets and the geometric deformation can have a profound effect on the ϵ_0 and Γ and subsequently on the measured $I - V$ characteristics. Thus, a statistical evaluation of the non-equilibrium state distribution is necessary for all relevant configurations, which contribute significantly to the single-level model parameters at each tip separation.

In order to sample the configuration space of the PEEB-S molecule clamped between the gold electrodes, we define a grid composed of 540 anchoring points on both facets as shown in Figure S7(a). The APs are separated by a distance of 0.476 Å and are located at a height of 3 Å above the facet. Those extending over the facet edge are curved towards electrode edges to maintain a distance of 3 Å to the edge (see Figure S7(b)). The distribution of the APs matches the local coordination symmetry of the facet (see Figure S7(c)). A pairwise selection of APs on left and right facets are utilized as placeholders for sulfur atoms of the PEEB-S molecule. The mismatch between the longitudinal dimension of the PEEB-S molecule and the separation between the APs is compensated by stretching or bending the molecular backbone of PEEB-S. Thus, the maximum number of configurations possible for a single tip-tip separation can amount up to 291 600 for every $S_{tip-tip}$. We consider values of $S_{tip-tip}$ in the range of 11.5 Å to 26.46 Å in steps of 0.825 Å.

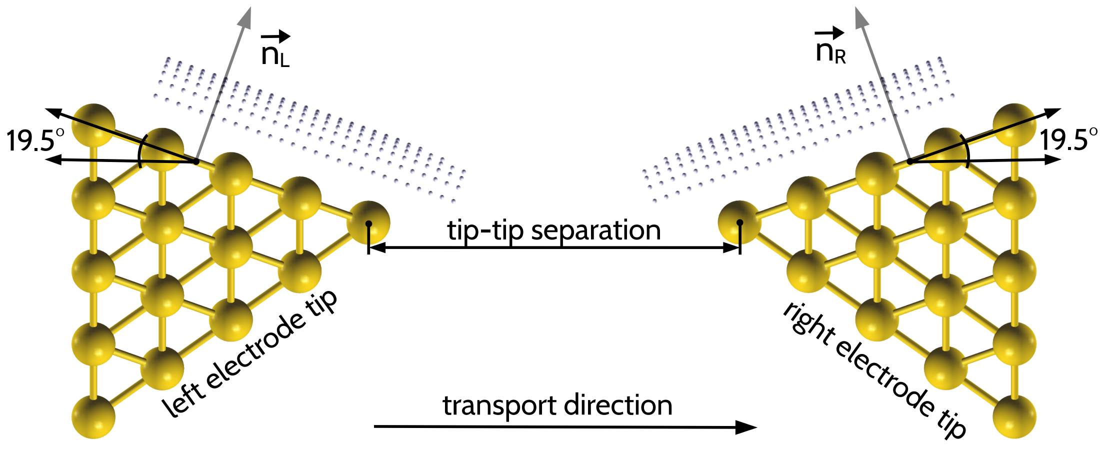

(a) anchoring points on left-/right facets

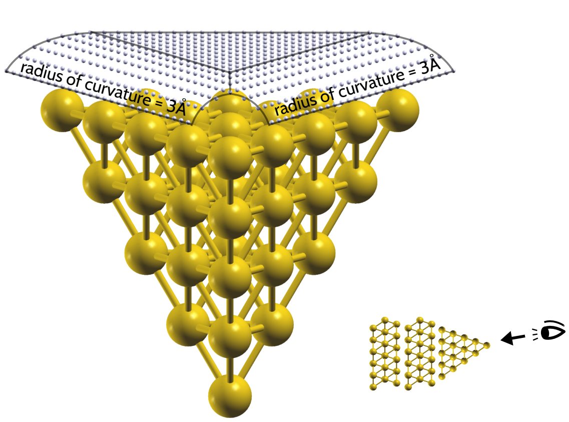

(b) Front view

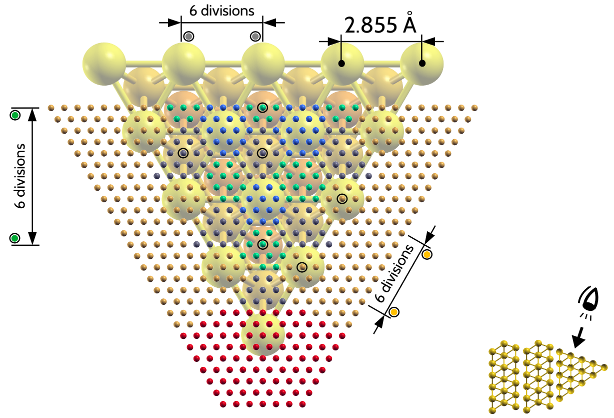

(c) Top view

Figure S7: (a) Anchoring points (APs) are shown using gray points for sulfur anchor points on gold facets. The tetrahedron-shaped tips have a base length of 4×2.855 Å. The facets are inclined at an angle of 19.5° to the transport direction z. The facet normal vectors ($\vec{n}_{L|R}$) are also shown using gray arrows. (b) The front view reveals the spacial distribution of the APs directly over the facet spaced evenly by 0.476 Å and of the APs protuding beyond the facet-edges curved towards the edges with a radius of curvature of 3 Å. (c) The top view along the facet normal vector reveals the equal spacing of the APs. The APs are divided into five subgroups: top, hcp, fcc, edge and tip, which are highlighted using the colored points: blue, dark gray, green, yellow and red, respectively. The distinction is performed based on the vicinity to either a high-symmetry site (top, fcc- and hcp- hollow site), or one of the edges or the tip atom nearest to the APs. The high-symmetry top, hcp-, and fcc-hollow sites of Au(111) are shown using yellow, dark yellow and dark orange colored spheres, respectively.

As mentioned above, geometric deformations can influence the ground state energy of PEEB-S molecule and induce orbital reordering as shown in Figure S8. Marginal level splitting is observed for unoccupied ($LUMO_{+2/+3}$) and occupied states ($HOMO_{-1/-2}$) for both compressive and tensile deformation. Level crossings are observed for unoccupied states ($LUMO_{+2/+3} \rightarrow LUMO/LUMO_{+1}$) for tensile deformation beyond 5 %. We include geometric deformations of the longitudinal dimension of the PEEB-S molecule in the range between 75 % and 105 %, where the level spacing HOMO to $HOMO_{-1/-2/-3}$ and LUMO to $LUMO_{+1/+2/+3}$ is sufficiently large to identify one dominant transport orbital and its SLM parameters. The subsequent geometry optimizations are performed in cluster mode with a constraint that allows the anchoring sulfur atoms to move along the facet normals. The constraint ensures, that the relaxed configurations are unique and the sampling of the configuration space is unbiased.[7–10] The configuration space of the relaxed geometries, acts as the basis for evaluating the (non-)equilibrium statistical weights of the single-level model parameters, in order to model diffusion and deformation processes during the measurement.

In the absence of deep local minima, the diffusion of the anchor atoms occurs on a timescale that is short with respect to the measurement time used in the experiments and thus configurations contribute to a thermodynamic mean according to the Boltzmann distribution. In the case of PEEB-S with thiol-anchoring groups anchored between gold electrodes, however, there exist deep local minima enclosed by ranges of high-energy configurations, which are unlikely to be overcome on the timescale of the experimental measurement. In order to evaluate also the contributions of geometrically restricted configurations to an experimentally measured mean, we model the thermal sampling of configurations by a random walk on the 4-dimensional configuration space described by the binding sites on both electrodes. Our approach is based on a master equation,[5] defining a transition matrix, which we use to propagate the probability density. The transition rates are the Metropolis probabilities[4] for neighboring configurations and zero elsewhere. Neighboring configurations in this sense are defined as geometries that differ in only one of the anchoring positions by a single grid spacing. Note that this process leads to the Boltzmann distribution asymptotically at infinite times. Hence, our general approach can be used to characterize both a non-equilibrium and an equilibrium system by assessing the slowdown in the approach speed to equilibrium. In a system geometrically restricted from reaching equilibrium, states in the tributary areas of deep local minima follow Boltzmann distributions locally as observed in Figure S7.

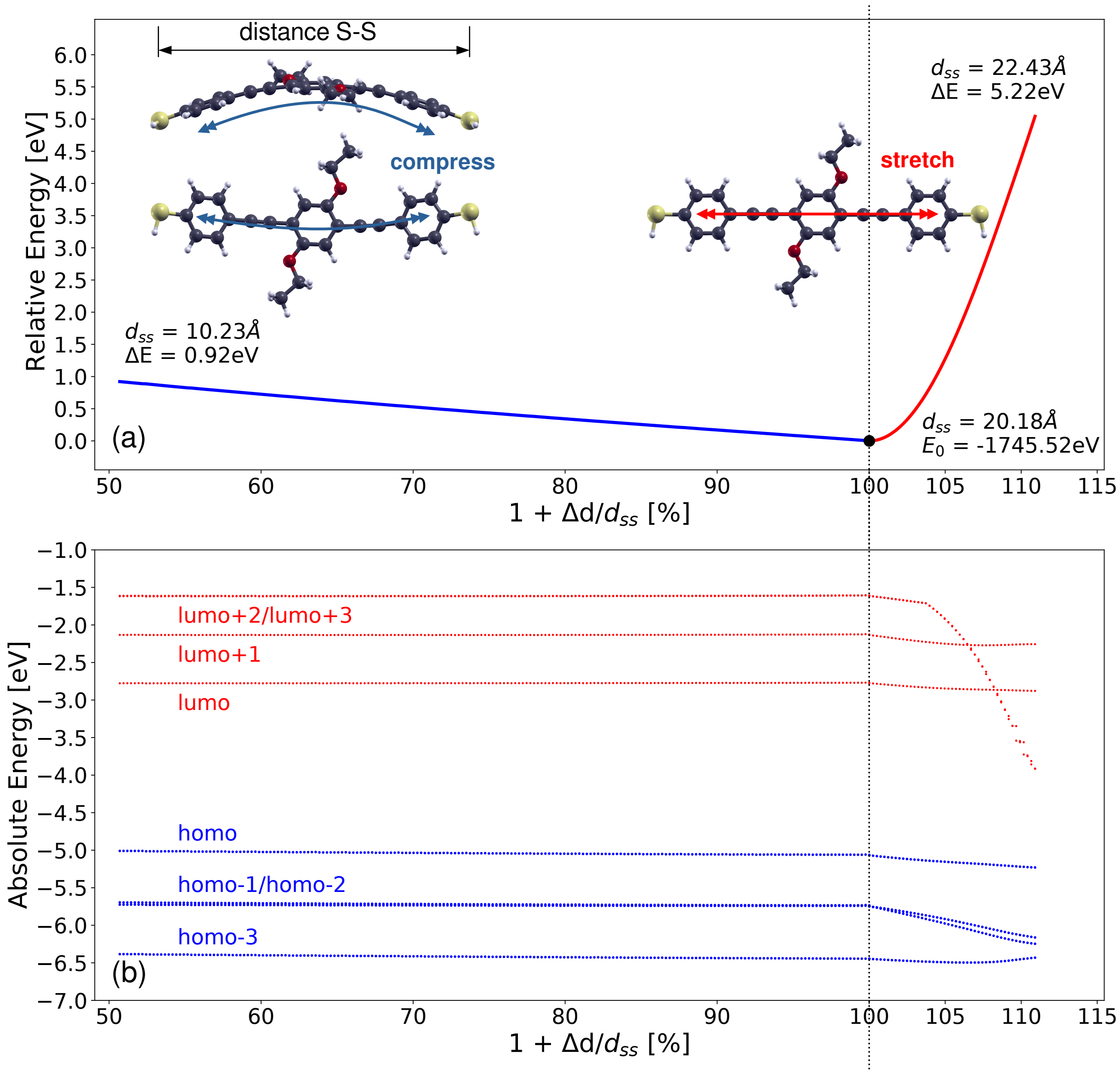

Figure S8: Compressive and tensile deformation of the PEEB-S molecule along the longitudinal dimension. (a) Deformation energy due to compressive/tensile strain - compressive strain induces a linear increase (blue line). The slope of the line amounts to 0.09 $^{\mathrm{eV}}/_{\mathrm{Å}}$. The molecule can counteract the compressive axial strain by distributing the force along the two remaining transversal axes. The dependency of the total energy with tensile strain follows Hooke's laws, indicated by the red parabola. The spring constant amounts to 2.06 $^{\mathrm{eV}}/_{\mathrm{Å}^2}$ (b) Level-crossing of the frontier molecular orbitals (HOMO and LUMO) upon deformation - marginal level splitting of frontier molecular orbitals (HOMO and LUMO) is observed for unoccupied ($\mathrm{LUMO}_{+2/+3}$) and occupied states ($\mathrm{HOMO}_{-1/-2}$) for both compressive and tensile deformation. Level crossings are observed for unoccupied states ($\mathrm{LUMO}_{+2/+3} \rightarrow \mathrm{LUMO}/\mathrm{LUMO}_{+1}$) for tensile deformation $\geq$ 7%.

S6: Mean Curvature

Mean curvature ($\overline{mC}$) of a PEEB-S molecule anchored between gold electrodes is shown in Figure S9. The atoms along the MB of a PEEB-S molecule are indexed in increasing order. The centers of the three phenyl rings of the PEEB-S molecule are determined by evaluating the center of mass of the remaining four carbon atoms not enumerated in the previous step. The anchoring sulfur atoms are also included as starting and ending point. The menger curvature[11] is determined for successive triplets starting at one of the anchoring sulfur atoms. The mean curvature ($\overline{mC}$) denotes the mean of menger curvatures for all the successive triplets.

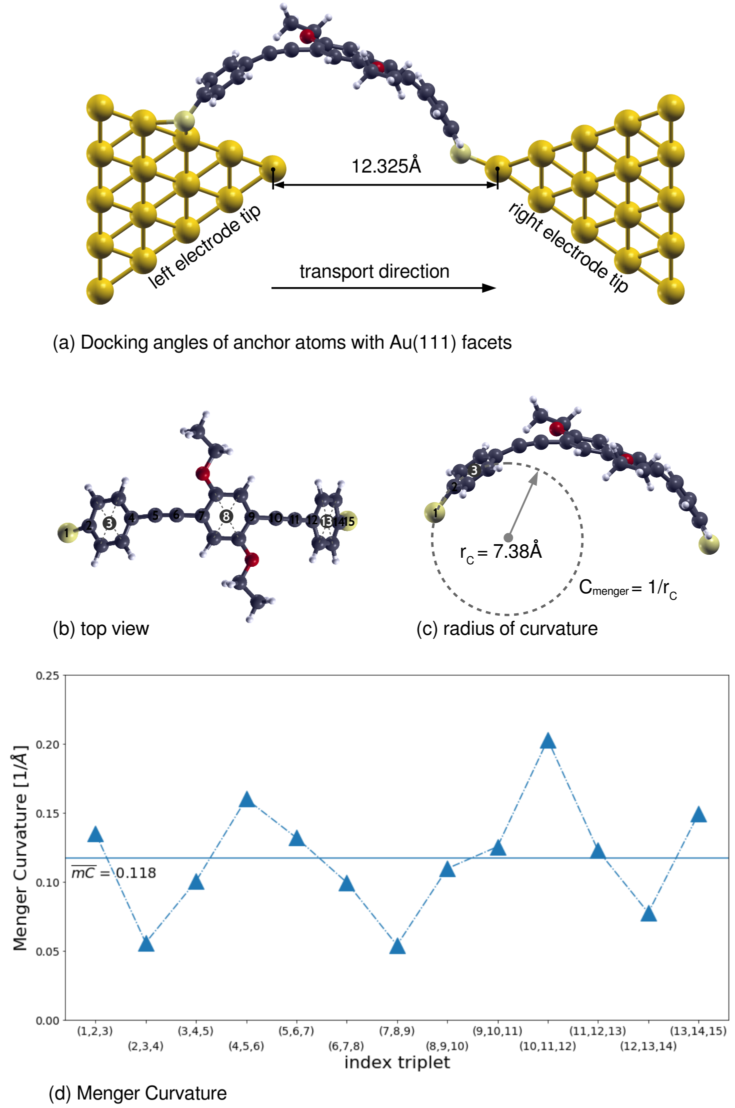

Figure S9: Mean curvature ($\overline{mC}$) of a single PEEB-S molecule between gold electrodes. (a) Top view of a representative configuration. The carbon atoms along the MB are enumerated. The centers of mass of the excluded four carbon atoms of the three phenyl rings are included, as well as the peripheral sulfur atoms. (b) The radius of curvature (r_C) for the index triplet $(1, 2, 3)$ is 7.38 Å. The r_C is inverse of the menger curvature (C_{menger}). The C_{menger} for a triplet of points (p_1, p_2, p_3) is given by $C_{menger}(p_1, p_2, p_3) = \frac{4A}{|p_1-p_2||p_2-p_3||p_3-p_1|}$, where A denotes the area of the triangle spanned by p_1, p_3 and p_3. (c) The C_{menger} is shown for all enumerated triplets of the representative configuration. Smaller values of C_{menger} are observed for the triplets (p_2, p_3, p_4), (p_7, p_8, p_9) and (p_{12}, p_{13}, p_{14}), which can be attributed to the stiffness/planarity of the phenyl rings. The mean curvature (mC) of the representative configuration amounts to 0.118 Å.

S7: Anchoring Angle

The left and right anchoring angles ($\angle_{LA}$ and $\angle_{RA}$) are determined using the bond vectors $\vec{n}_{L-SC}$, $\vec{n}_{R-SC}$ and the facet normal vectors $\vec{n}_{L}$, $\vec{n}_{R}$, respectively. We denote the bond vectors spanned between the left and right anchoring sulfur atom and the ortho-/para-carbon atom of the left-/right-peripheral phenyl ring (PPR) using $[L|R]_{S}C$, respectively. The bond vectors are extended between the anchoring sulfur atoms and the nearest carbon atom in the peripheral phenyl rings directed towards the carbon atoms. The normal vectors play an essential role during the relaxation simulations. The sulfur atoms, once placed on specific grid points on the left and right facets, are only allowed to move along the left and right normal, respectively.

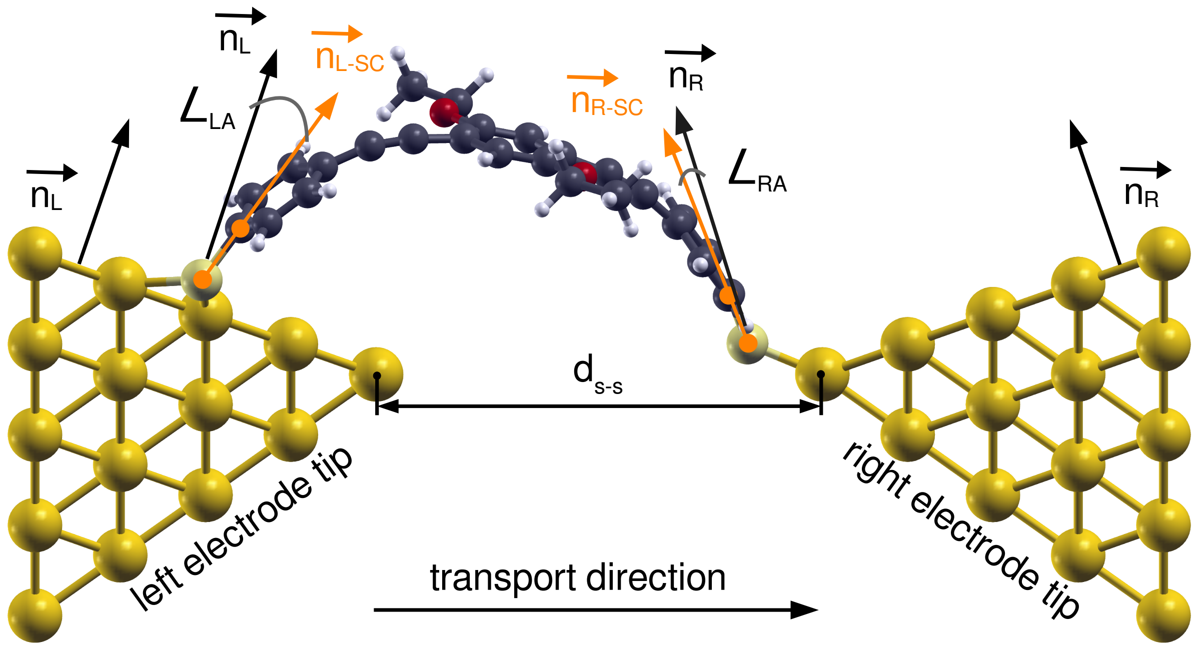

Figure S10: The left and right anchoring angles $\angle_{LA}$ and $\angle_{RA}$, of a single PEEB-S molecule between gold electrodes. The anchoring angles are calculated using two vectors: $\vec{n}_{[L|R]-SC}$ and $\vec{n}_{[L|R]}$. $\vec{n}_{[L|R]-SC}$, shown using orange arrows, correspond to the bond vectors spanned between the left and right anchoring sulfur atom and the ortho-/para-carbon atom of the left-/right-PPR. $\vec{n}_{[L|R]}$, shown using black arrows, correspond to the $\vec{n}_{facet}$. The anchoring angles, $\angle_{LA}$ and $\angle_{RA}$, are the angles between the corresponding left and right vector pairs $(\vec{n}_{[L|R]-SC} \mid \vec{n}_{[L|R]})$. The distance between the centers of the tip atoms of the left and right gold electrodes is given by d_{s-s}. For the representative configuration shown in the figure the values for $\angle_{LA}$, $\angle_{RA}$ and d_{s-s} are 34.73°, 5.61° and 12.33 Å, respectively.

S8: Curve Fitting: Transmission Function

ϵ_0 and $\Gamma_{min|max}$ can be evaluated by fitting the single-level model to the transmission function $T(E)$ calculated using equilibrium Green's function (EGF) (see SI Section S1). The fitting results are shown for 8 configurations sorted according to their random-walk weights for the $S_{tip-tip} = 12.33\,\text{Å}$ in Figure S11. We adopt asymmetric coupling of the dominant transport orbital to the metallic electrodes. We identify in the first step the peaks in the transmission function and choose the peak closest to the Fermi level. The x position of this peak can be used to define the constraints on ϵ_0. The lower and upper bound for ϵ_0 are defined as $x \pm 0.2\,\text{eV}$, respectively. The lower and upper bound for $\Gamma_{min|max}$ are defined as $0.0\,\text{eV}$ and $1.0\,\text{eV}$. We use the trust region reflective algorithm[12] with bounds for curve-fitting. In our analysis of the theoretically determined stretch evolution of the SLM parameters $(\overline{\epsilon}_0^T|\overline{\Gamma}^T)$, we include upto 1000 configurations with the highest random-walk weight for every $S_{tip-tip}$. For a subset of these configurations, the transmission function that cannot be fitted to the single level model (see Figure S11 configuration 1). We do not reject the subset, since the associated configurations are characterized by optimal anchoring possibility between the metallic electrodes and our fitting procedure underestimates the value of $\Gamma = {}^{\Gamma_{min}+\Gamma_{max}}/_{2}$.

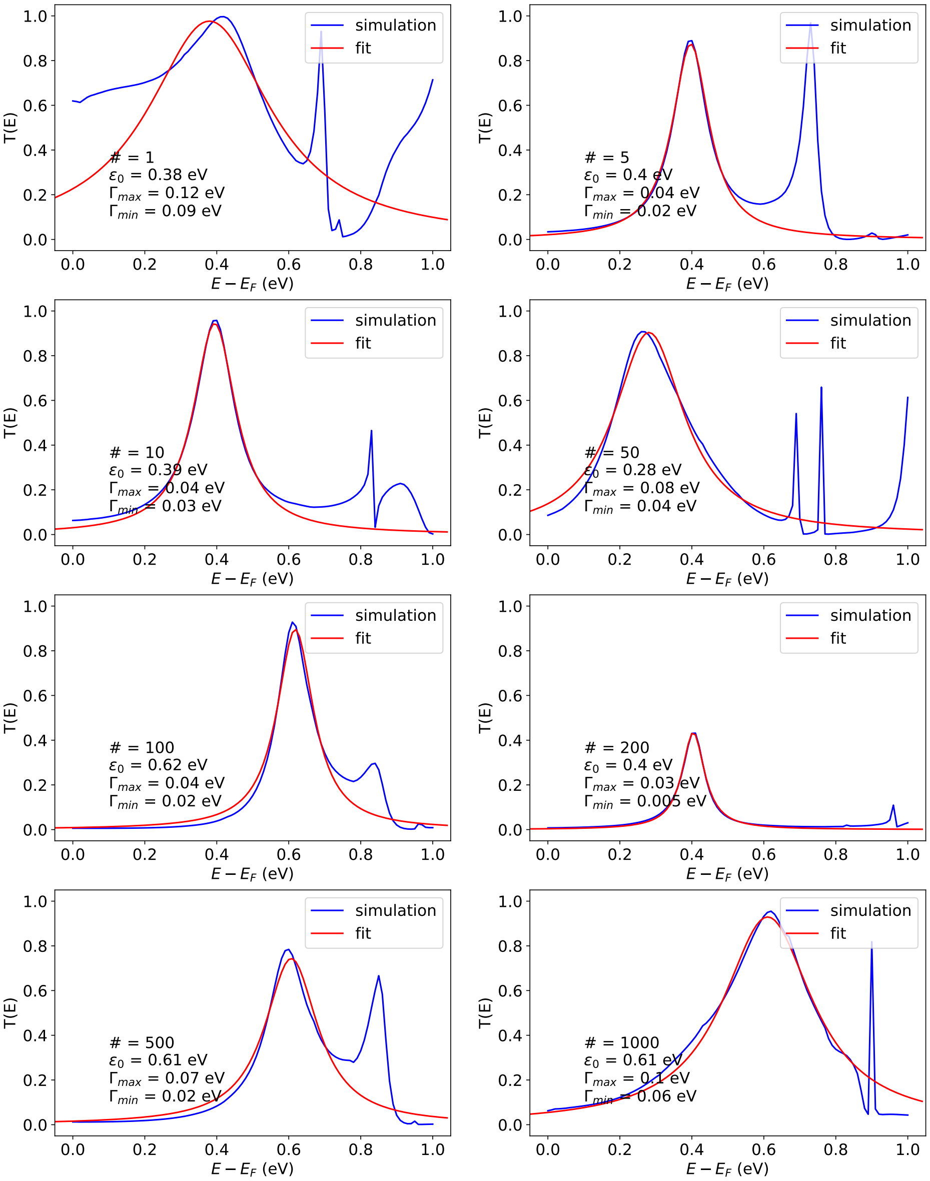

Figure S11: Evaluating the single-level model parameters (ϵ_0 and $\Gamma_{min|max}$) assuming asymmetric coupling of the dominant transport orbital to the metallic electrodes. 8 configurations are chosen for the $S_{tip-tip} = 12.33\,\text{Å}$. The transmission function ($T(E)$) at zero-bias (blue curves) are calculated using the EGF formalism. The ϵ_0 and Γ are evaluated by fitting the SLM (red curve), assuming asymmetric coupling of the transport orbital to the metallic electrode.

S9: Real-space Projection Γ_{mean}

In Figures S12 and S13, the real space projection of Γ_{mean} is shown for different number of configurations for the $S_{tip-tip} = 12.33$ Å. The side and top view of the configuration with the strongest electronic coupling is shown in figure S12(a) and (b), respectively. The strongest coupling at the $S_{tip-tip} = 12.33$ Å occurs for anchoring at bridge sites (see Figure S12(c)) along the Au(111) facet edges of both the left and right electrodes.

In Figure S13, the real-space projection of Γ_{mean} is shown for different number of configurations for the $S_{tip-tip} = 12.33$ Å. The two dominating configurations with strong electronic coupling are anchored at bridge sites along the Au(111) facet edges.

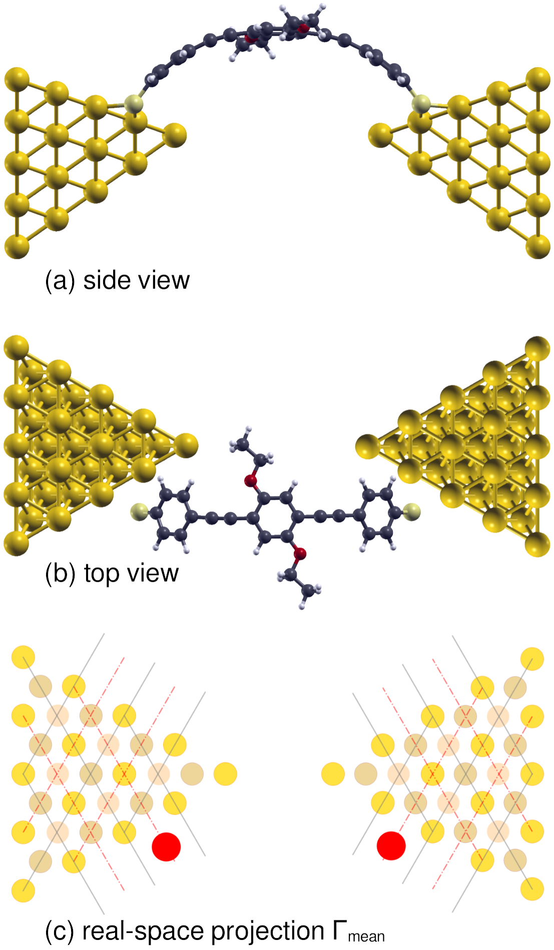

Figure S12: Real-space projection of Γ_{mean} (a) side view and (b) top view (not to scale) of the configuration with strongest coupling for the $S_{tip-tip} = 12.33$ Å. (c) The value of Γ_{mean} is projected in form of red colored circles in place of the anchoring sulfur atoms on the left and right electrodes in top view. The gold atoms of the electrodes are colored to depict the top, hcp-hollow and fcc-hollow sites using yellow, brown and salmon colored circles respectively. The solid gray and dotted red lines are shown to differentiate between anchoring of the sulfur atoms at top and bridge sites along the Au(111) facet edges of both the left and right electrodes.

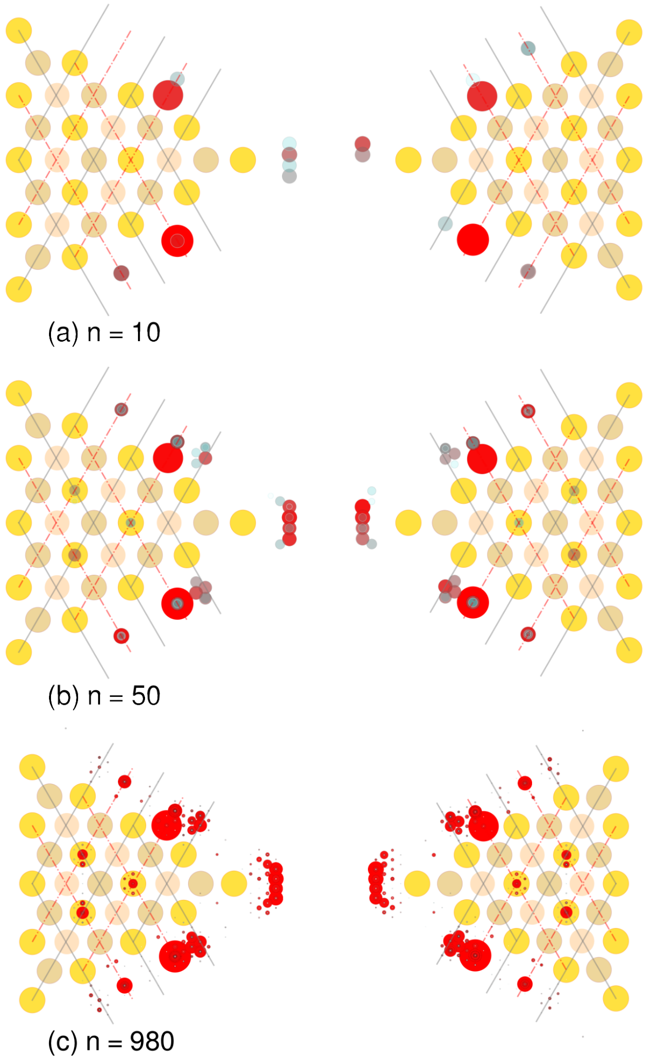

Figure S13: Real-space projection of Γ_{mean} for different number of configurations for the $S_{tip-tip}$ = 12.33 Å. The number of configurations shown are (a) 10 , (b) 50 and (c) 980 . The strength of electronic coupling is represented from higher to lower values using a color scale spanning from red to gray, respectively. The strength of electronic coupling is also represented using colered circles of varying sizes. The large/small sized circle represent strong/weak electronic coupling respectively.

S10: Cross Correlation

In Figure S14, the cross correlation plot is shown between $S_{tip-tip}$, $\overline{\epsilon}_0$, $\overline{\Gamma}_{max}$, $\overline{\Gamma}_{min}$, $\overline{mC}$, $\angle_{LA}$ - left anchoring angle, $\angle_{RA}$ - right anchoring angle and the anchoring positions (tip, edge, top, fcc- and hcp-hollow sites). We evaluate pearson standard correlation coefficients.[13]

A strong correlation is observed between $\overline{\epsilon}_0$ and, $\angle_{LA}$ and $\angle_{RA}$, as well as between $\overline{\epsilon}_0$ and the tip anchoring positions. This suggest, that for an optimal anchoring angles and tip anchoring position, the HOMO-LUMO gap approaches the unperturbed molecular HOMO-LUMO gap. Similarly, a strong correlation is observed between $\overline{\Gamma}_{max}$, $\overline{\Gamma}_{min}$ and the $\overline{mC}$. This suggests that a planar configuration of the molecule is associated with a strong electronic coupling between the transport channel and the metallic states.

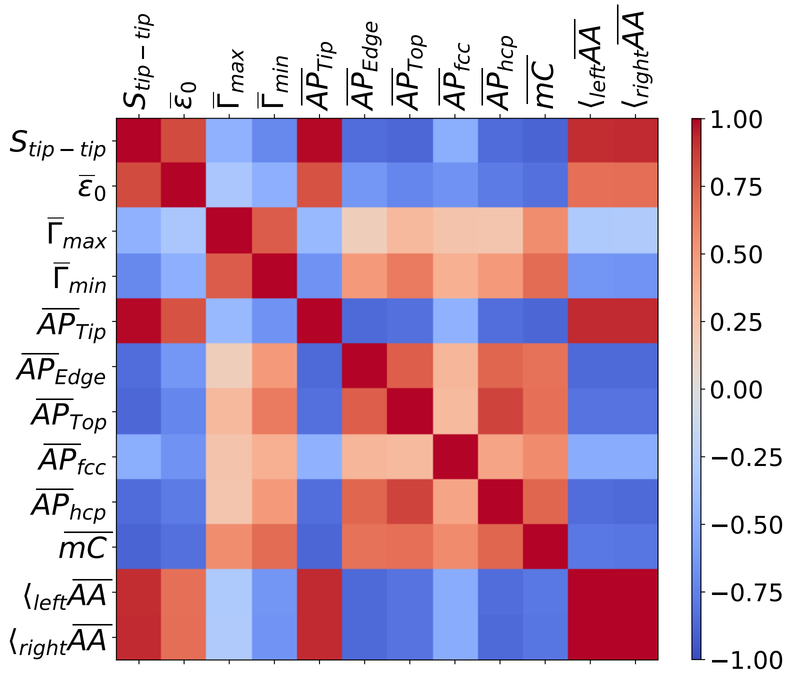

Figure S14: Cross-correlation plot between $S_{tip-tip}$, $\overline{\epsilon}_0$, $\overline{\Gamma}_{max}$, $\overline{\Gamma}_{min}$, $\overline{mC}$, $\angle_{LA}$ and $\angle_{RA}$ and the anchoring positions (tip, edge, top, fcc- and hcp-hollow sites). A strong/weak correlation corresponds to the values $1\,/-1$, respectively.

S11: Evolution of Single-Level Model Parameters (ϵ_0 and $\Gamma_{min|max}$)

The experimentally measured stretch evolution of the SLM parameters ($\epsilon_0^E|\Gamma^E$) and the $\bar{\epsilon}_0^T|\overline{\Gamma}^T$ are shown in Figure S15. The notation E and T indicate

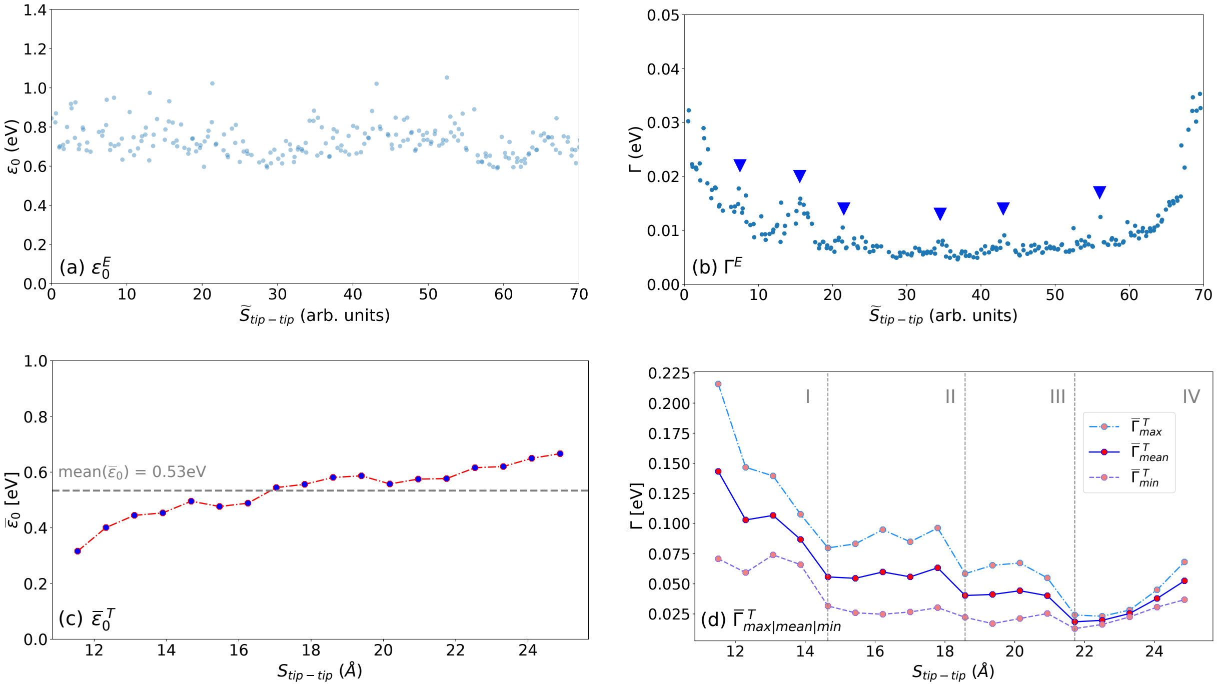

Figure S15: Experimentally measured stretch evolution of the SLM parameters ($\epsilon_0^E|\Gamma^E$) for opening measurement of Co-Salen and theoretically determined stretch evolution of the SLM parameters ($\bar{\epsilon}_0^T|\overline{\Gamma}^T$), which are calculated for 1000 thermodynamically most relevant configurations for individual tip-tip separation ($S_{tip-tip}$). (a) ϵ_0^E - the data points scatter between 0.58 eV and 1.05 eV. The mean value $\bar{\epsilon}_0$ is 0.73 eV. The standard deviation amounts to 0.0826 eV. (b) Γ^E - the data points with local maxima, which are marked using dark blue arrows. (c) $\bar{\epsilon}_0^T$ - an overall rising trend is visible. The mean value of $\bar{\epsilon}_0$ is 0.53 eV. (d) $\overline{\Gamma}^T_{max}$, $\overline{\Gamma}^T_{mean}$ and $\overline{\Gamma}^T_{mean}$ all reveal a falling trend for $S_{tip-tip}$ interval (11.51 Å $< S_{tip-tip} <$ 21.72 Å) and a rising trend for $S_{tip-tip} >$ 21.72 Å. Local maxima are visible for $\overline{\Gamma}^T_{mean}$ at $S_{tip-tip}$: 13.08 Å, 17.79 Å and 21.72 Å, respectively.

The experimentally measured stretch evolution of ϵ_0 (ϵ_0^E) does not reveal any trends or features (see Figure S15(a)). The data points for ϵ_0 oscillate about the mean value of 0.72 eV in the range of 0.6 eV to 1.0 eV. For reasons of completeness, we show the theoretically determined stretch evolution of $\bar{\epsilon}_0$ ($\bar{\epsilon}_0^T$) in Figure S15(c). Since we choose PEEB-S over dithiolated-N,N'-bis(5-ethynylbenzenethiol-salicylidene)ethylenediamine cobalt complex (Co-Salen-S) to reduce computational effort, we cannot directly compare the stretch evolution of ϵ_0 measured in experiments with theory and reserve a detailed comparison of the stretch evolution of ϵ_0 at low temperatures for future studies. In contrast, a comparison of the stretch evolution of

electronic coupling is feasible, since both molecules bear thiol anchoring groups and exhibit similar binding properties to gold electrodes. In the initial opening phase, we notice a falling trend in Γ^E and $\overline{\Gamma}^T$, which flattens out for the larger part of the opening curve, only to be superseded by a rising trend in the final phase before break-off. Both, Γ^E and $\overline{\Gamma}^T$ reveal distinct local maxima.

S12: Selected configurations

In Figure S16, selected configurations are shown with anchoring between (a) tip and facet, (b) edge and edge, (c) tip and edge, and (d) tip and tip.

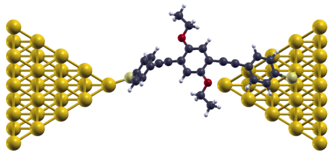

(a) tip-facet

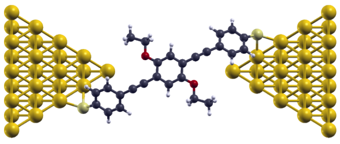

(b) edge-edge

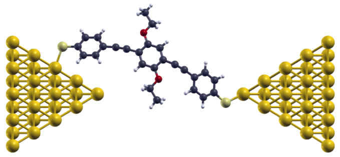

(c) tip-edge

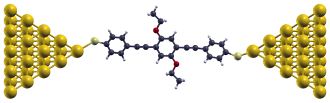

(d) tip-tip

Figure S16: Selected configurations with anchoring positions (a) tip-facet, (b) edge-edge, (c) tip-edge, and (d) tip-tip.

References

(1) Kim, Y.; Pietsch, T.; Erbe, A.; Belzig, W.; Scheer, E. Benzenedithiol: A Broad-Range Single-Channel Molecular Conductor. *Nano Letters* **2011**, *11*, 3734–3738.

(2) Kilibarda, F.; Strobel, A.; Sendler, T.; Wieser, M.; Mortensen, M.; Trads, J. B.; Helm, M.; Kerbusch, J.; Scheer, E.; Gemming, S.; Gothelf, K. V.; Erbe, A. Single-Molecule Doping: Conductance Changed By Transition Metal Centers in Salen Molecules. *Advanced Electronic Materials* **2021**, *7*, 2100252.

(3) Lo, K. H.; Christensen, R. M.; Wu, E. M. A High-Order Theory of Plate Deformation—Part 1: Homogeneous Plates. *Journal of Applied Mechanics* **1977**, *44*, 663–668.

(4) Metropolis, N.; Rosenbluth, A. W.; Rosenbluth, M. N.; Teller, A. H.; Teller, E. Equation of State Calculations by Fast Computing Machines. *The Journal of Chemical Physics* **1953**, *21*, 1087–1092.

(5) Van Kampen, N. In *Stochastic Processes in Physics and Chemistry (Third Edition)*, third edition ed.; Van Kampen, N., Ed.; North-Holland Personal Library; Elsevier: Amsterdam, 2007; pp 96–133.

(6) Love, J. C.; Estroff, L. A.; Kriebel, J. K.; Nuzzo, R. G.; Whitesides, G. M. Self-Assembled Monolayers of Thiolates on Metals as a Form of Nanotechnology. *Chemical Reviews* **2005**, *105*, 1103–1170.

(7) Ferrenberg, A. M.; Landau, D. P.; Binder, K. Statistical and systematic errors in Monte Carlo sampling. *Journal of Statistical Physics* **1991**, *63*, 867–882.

(8) Landau, D. P. Finite-size behavior of the Ising square lattice. *Phys. Rev. B* **1976**, *13*, 2997–3011.

(9) Tokdar, S. T.; Kass, R. E. Importance sampling: a review. *WIREs Computational Statistics* **2010**, *2*, 54–60.

(10) Kunze, T.; Gemming, S.; Numazawa, S.; Schreiber, M. Low-temperature modeling for degenerate and frustrated Heisenberg systems with anisotropy. *Computer Physics Communications* **2010**, *181*, 806 – 812.

(11) Leger, J. C. Menger Curvature and Rectifiability. *Annals of Mathematics* **1999**, *149*, 831–869.

(12) Sorensen, D. C. Newton's Method with a Model Trust Region Modification. *SIAM Journal on Numerical Analysis* **1982**, *19*, 409–426.

(13) Rahman, N. A. *A Course in Theoretical Statistics*; Charles Griffin and Company, 1968.
