# Supplementary material for: Stretch Evolution of Electronic Coupling of the Thiophenyl Anchoring Group with Gold in Mechanically Controllable Break Junctions
Source: J Phys Chem Lett. 2023 Jun 15;14(24):5709–17. doi: 10.1021/acs.jpclett.3c00370 (PMC10291638; doi:10.1021/acs.jpclett.3c00370)
Supplement: Supplementary file 2 — jz3c00370_si_003.pdf [file jz3c00370_si_003.pdf]

jz-2023-00370f.R1

Name: Peer Review Information for "Stretch Evolution of Electronic Coupling of the Thiophenyl Anchoring Group with Gold in Mechanically Controllable Break Junctions"

First Round of Reviewer Comments

Reviewer: 1

Comments to the Author

The authors study evolution of electronic coupling (Γ) during the stretching process of single-molecule junctions. Experiments were performed for N,N-Bis(5-ethynylbenzenethiol-salicylidene)ethylenediamine cobalt complex (Co-Salen) using the MCBJ technique and evolution of Γ was analyzed based on the resonant level model. Theoretical simulations were done for thiol-terminated 1,4-bis(phenylethynyl)-2,5-bis(ethoxy)benzene (PEEB) based on transport calculations in combination with statistical averaging approach. They experimentally revealed peculiar oscillations of Γ during the junction stretching process and found a link between the evolution of Γ in the MCBJ measurements for Co-Salen and the microscopic structure of PEEB obtained from the theoretical simulations.

I think the authors report a new, high-throughput dynamic simulation approach that models many thermodynamically relevant configurations that can form in single-molecule transport experiments. This manuscript is a nice work on an interesting topic in the molecular electronics community, but there are several points that need to be addressed. I recommend that this manuscript be published with major revisions or presented elsewhere as a theoretical regular paper.

Comment 1

>The metal complex (Co-Salen) was studied in the experiments, while the theoretical simulation was performed for the OPE3 derivative (PEEP). Co-Salen has different mechanical and electronic properties from PEEP because of the metal center and longer molecular length. To compare experimental and theoretical results, the author should present experimental data on PEEP or theoretical data on Co-Salen.

Comment 2

p. 11, last sentence "Due to the elongation of the peripheral PEEB … the central metal complex has minimal effect on the stretch evolution of Γ."

> Please explain the sentence more clearly. I think that the metal center will have a non-negligible impact on the stretching evolution of Γ for Co-Salen.

Comment 3

> As I understand it, the theoretical simulation determines a value of Γ by statistical averaging of Γ values for the probable junction models, while the experiments fit a single I-V curve to the resonance level model to obtain a value of Γ. I am not sure a single I-V curve for a junction with structural fluctuations and transitions can be fitted to the simple resonance model. Is it possible to evaluate the validity of the statistical averaging approach using alternative model experimental systems? Or has its validity already been demonstrated? In this connection, please explain in detail the procedure for the I-V curve fitting in SI. See also the comment 5.

Comment 4

>Fig. 3c and Fig. S4 are missing in the manuscript pdf files.

Comment 5

> Details of the experiment are not well described.

>For example, stretch length and speed of the junction are not mentioned.

>Measured I-V curves and results of curve fitting are not shown.

>Equation (1) is not described in the cited reference [3] (SI, p. 2).

Minor

6. Fig 5b, caption

>dithiolated? 1,4-bis(phenylethynyl)-2,5-bis(ethoxy)benzene

7. Figure S10

>How do you determine Γmax and Γmin? I think Γ(=Γmax+Γmin?) corresponds to half width at half maximum of the Lorentzian peaks in Fig. S10.

Some of the terms "maxima" and "minima" in the text seemed to be used in a confusing manner.

Reviewer: 2

Comments to the Author

The paper of Lokamani et al. presents a computational study of the electronic coupling of a molecule thiol groups to gold. The results are compared to MCBJ measurements. The main novelty of the study is the theoretical approach used, relies on sampling and weighing possible configurations of the molecule. I think this approach is a reasonable alternative to analyzing geometries from molecular dynamics trajectories. The approach described here is novel and could be a significant contribution. I think the paper could be published, however some changes are needed first.

The procedure used for generating and evaluating trajectories should be described in more detail. If it, after all, the main contribution of the paper but its description is displaced instead to the supplemental section. The authors should specify here the advantages of their proposed approach over MD trajectories.

It is sometimes hard to follow the discussion of the trends in figure 2 without seeing the structure of the scattering region. Can the authors add snapshots in the supplementary section? Why are configurations where S is adsorbed on the planar part of the pyramid relevant at certain distances (figure 4e) but neither before nor after?

Why does Gamma increase when the junction is breaking? I would expect it do decrease as the contact is broken.

Supplemental figure 10 shows some examples of T(E) and their fits. Many details of the fit are missing. What range are they fitted over? Sub-figure 1 (#1) is a terrible fit that should be rejected. Is there any goodness-of-fit criteria applied or are all fits (such as #1) included regardless of their quality? What do the data look like on a logarithmic scale?

Reviewer: 3

Comments to the Author

The manuscript of M. Lokamani et al. describes a theoretical model of the electronic molecule-electrode coupling evolution along the stretch of a molecular junction based on thiolate PEEB compound between gold electrodes. It explores the contribution of three geometrical descriptors: the anchoring sites, the curvature of the molecular backbone and the thiol-electrode binding angle. The theoretical results are related to some examples of the evolution of the parameter Gamma obtained from fitting experimental current-voltage (IV) curves recorded during the stretch of a Salen compound / gold molecular junction to the single level model (SLM). In my opinion, this work suggest interesting contributions to the electronic coupling in a molecular junction in an exhaustive and comprehensive way and I think the manuscript should be published. However, there are some points that the authors must address before publication:

Regarding the experimental section:

1) The experimental section in the manuscript is very brief. Relevant information is missing. For instance the authors mention that 700 stretching cycles were studied, but give not statistics in how many of them reproduce the behavior of interest during the stretching: an initial decrease of Gamma followed by a somehow flat region with individual peaks and finally a new Gamma increase. Was this the main observed behavior or a marginal one? Which was the main observed behavior in the latter case? (only three stretching examples are given in the manuscript). Although the fact the behavior could be not frequent would not mean that it is not interesting, this information should be given in the manuscript.

2) Additionally, how fast were the IV curves recorded in the experiment? The system was given time to relax after each stop before recording the IV curves? Were both IV curves recorded at a given point identical?

3) The authors should give examples of the 'detected molecular signatures' that they used as evidence that a molecular junction had formed, as well as the examples of experimental IV curves and their fits at least in the supporting information (SI) document (fits to the theoretical T(E) curve are indeed included in this document). In particular, which expression was fitted to the experimental IV curves? Only one Gamma is given per position. Does this mean that only symmetric fits were considered, or this Gamma is the average of Gamma_min and Gamma_max?

4) An experimental Gamma decrease reflects mainly a conductance decrease. Can the decreasing direct tunneling contribution account for the experimental Gamma decrease during the initial part of the stretching?

5) Why was this particular Salen compound used in this work when the theoretical calculations were done for a simpler PEEB compound? Were experiments done with PEEB as well? If so, were Gamma showing the same evolution? These points should be clarified in the manuscript.

6) The magnitude d_0 mentioned at the end of section 1 of the SI document is not defined. What does it mean?

Regarding the model:

7) What magnitude is exactly quantified and represented in Figure 2(a) as AP? Is it the probability weight of each configuration? Which are the units? Please clarify in the manuscript.

8) In the theoretical section, what does it mean 'thermodynamical weights according to individual energies and involved energy landscapes'? Please clarify in the manuscript.

9) In the description of Figure 13, the authors describe a 'strong correlation' of eplison_0 with the left and right anchoring angles but that is not observed in Figure 13. First, the intersection of these magnitudes is blue, so there is a negative correlation or anticorrelation between them. Then, the colors is quite light pointing to a light or mild anticorrelation. Similarly, the intersection between the Gammas and mC presents a very light color but the text describes a strong correlation between them. Other positive and negative correlations are stronger in the figure but not highlighted. Also in this figure, why the correlation of the right and left angles with other magnitudes are different?

10) In particular in the same Figure 13, S_tip-tip has a negative correlation with AP_tip which puzzles me: as I understand it, for high S_tip-tip the tip-tip configuration is dominant, shouldn't it be a strong positive correlation between these magnitudes? Which is the definition of correlation used in this work? Please clarify.

11) Other comments:

- In my opinion the manuscript is not well structured and results difficult to read. The experimental and theoretical sections are at the end of the document, but the results section cannot read or understood without reading them first as not summary or explanation of what has been done in presented in this section. No even the magnitudes as Gamma_min and Gamma_max for instance are defined. Also part of the essential information to understand the results is presented in the SI document. I understand the need to be brief in a Letter, but I consider that the text should be consistent and easy to read without continuous going backs and forwards.

- In particular, the acronyms are not properly introduced: SLM is only introduced in Figure 1 caption, SCC-DFTB in the conclusions or PEEB is introduced after being used several times.

- The name of the first author is missing.

- References to figures are sometimes confusing as those of the SI document are not numbered as Figure SX.

- In section 7 of the SI document (Figure 9): two names are used for the same magnitude: n_(SL-C) and n_(L-SC). I guess SC accounts for sulfur-carbon, but please define.

Author's Response to Peer Review Comments:

Response to referee report

"Stretch Evolution of Electronic Coupling of the Thiophenyl Anchoring Group with Gold in Mechanically Controllable Break Junctions"

Lokamani, Filip Kilibarda, Florian Günther, Jeffrey Kelling, Alexander Strobel, Peter Zahn, Guido Juckeland, Kurt Gothelf, Elke Scheer, Sibylle Gemming and Artur Erbe
April 14, 2023

Dear Editors,

we thank the reviewers for assessing our manuscript and for providing constructive feedback. In response, we have revised our manuscript carefully. Our detailed response to the individual points raised by the reviewers is provided below.

We hope our revised manuscript is now suitable for publication.
Sincerely,
Lokamani
Filip Kilibarda
Florian Günther
Jeffrey Kelling
Alexander Strobel
Peter Zahn
Guido Juckeland
Kurt Gothelf
Elke Scheer
Sibylle Gemming
Artur Erbe

Referee 1
Recommendation: This paper is probably publishable, but major revision is needed; I do not need to see future revisions.
Comments:
The authors study evolution of electronic coupling (Γ) during the stretching process of single-molecule junctions. Experiments were performed for N,N-Bis(5-ethynylbenzenethiol-salicylidene)ethylenediamine cobalt complex (Co-Salen) using the MCBJ technique and evolution of Γ was analyzed based on the resonant level model. Theoretical simulations were done for thiol-terminated 1,4-bis(phenylethynyl)-2,5-bis(ethoxy)benzene (PEEB) based on transport calculations in combination with statistical averaging approach. They experimentally revealed peculiar oscillations of Γ during the junction stretching process and found a link between the evolution of Γ in the MCBJ measurements for Co-Salen and the microscopic structure of PEEB obtained from the theoretical simulations.
I think the authors report a new, high-throughput dynamic simulation approach that models many thermodynamically relevant configurations that can form in single-molecule transport experiments. This manuscript is a nice work on an interesting topic in the molecular electronics community, but there are several points that need to be addressed. I recommend that this manuscript be published with major revisions or presented elsewhere as a theoretical regular paper.

Comment 1
>The metal complex (Co-Salen) was studied in the experiments, while the theoretical simulation was performed for the OPE3 derivative (PEEP). Co-Salen has different mechanical and electronic properties from PEEP because of the metal center and longer molecular length. To compare experimental and theoretical results, the author should present experimental data on PEEP or theoretical data on Co-Salen.
Answer 1:
We thank the reviewer for pointing out this important aspect. At tip-tip separations of the order of the dimension of the PEEB molecules, tunneling currents between the tip electrodes dominate over the molecular signature. As a result, stable measurements for determining the stretch evolution of PEEB were not possible. We have mentioned the reason for the comparison of different molecules in the experiments and theoretical analysis in the revised manuscript.
(Main manuscript page 6 last paragraph)

Comment 2
p. 11, last sentence “Due to the elongation of the peripheral PEEB … the central metal complex has minimal effect on the stretch evolution of Γ.”
> Please explain the sentence more clearly. I think that the metal center will have a non-negligible impact on the stretching evolution of Γ for Co-Salen.
Answer 2:
We have changed the sentence:
The influence of the central metal complex on the stretch evolution of Γ can be neglected due to the length of the peripheral PEEB moieties.
(Main manuscript page 7 first paragraph)

Comment 3
> As I understand it, the theoretical simulation determines a value of Γ by statistical averaging of Γ values for the probable junction models, while the experiments fit a single I-V curve to the resonance level model to obtain a value of Γ. I am not sure a single I-V curve for a junction with structural fluctuations and transitions can be fitted to the simple resonance model. Is it possible to evaluate the validity of the statistical averaging approach using alternative model experimental systems? Or has its validity already been demonstrated? In this connection, please explain in detail the procedure for the I-V curve fitting in SI.

See also the comment 5.
Answer 3:
The validity of the statistical averaging approach has been demonstrated in previous works

[1] L. A. Zotti, T. Kirchner, J.-C. Cuevas, F. Pauly, T. Huhn, E. Scheer, and A. Erbe, *Revealing the Role of Anchoring Groups in the Electrical Conduction Through Single-Molecule Junctions*, Small **6**, 1529 (2010).
[2] E. H. Huisman, C. M. Guedon, B. J. van Wees, and S. J. van der Molen, *Interpretation of Transition Voltage Spectroscopy*, Nano Lett **9**, 3909 (2009).
[3] P. Gehring, J. M. Thijssen, and H. S. J. van der Zant, *Single-Molecule Quantum-Transport Phenomena in Break Junctions*, Nat Rev Phys **1**, 6 (2019).

We have added the details of the curve-fitting procedure in the SI and added a new section on curve-fitting for the experimental measurements.
(see SI Section S1 and S8).

Comment 4
>Fig. 3c and Fig. S4 are missing in the manuscript pdf files.
Answer 4:
We have made necessary corrections in the manuscript.

Comment 5
> Details of the experiment are not well described.
>For example, stretch length and speed of the junction are not mentioned.
>Measured I-V curves and results of curve fitting are not shown.
>Equation (1) is not described in the cited reference [3] (SI, p. 2).
Answer 5:
We have added details of the experiments including speed of the junction. We have also included a new section on the measured I-V curves and the results of the curve fitting. We thank the reviewer for pointing out that the Equation (1) is not described in the cited reference [3]. We have added the modified equation we use in our curve-fitting routines in the manuscript.
(Main manuscript page 5 lower half, SI Section S1)

Minor
6. Fig 5b, caption
>dithiolated? 1,4-bis(phenylethynyl)-2,5-bis(ethoxy)benzene
Answer 6:
We have made necessary corrections in the manuscript. We have renamed the molecules accordingly.

7. Figure S10
>How do you determine Γ_{max} and Γ_{min}? I think Γ(=Γ_{max}+Γ_{min}?) corresponds to half width at half maximum of the Lorentzian peaks in Fig. S10.
Answer 7:
We define Γ=Γ_{max}+Γ_{min} as described in the manuscript. We determine Γ_{max} and Γ_{min} using our curve-fitting routines both in the experiments and from simulations. We have included a new section on curve-fitting of the measured IV-curves in the SI.
(SI section 8)

Some of the terms "maxima" and "minima" in the text seemed to be used in a confusing manner.
Answer:

We have revised our manuscript and use “peaks” and “minima” instead.

Additional Questions:
Urgency: Moderate
Significance: High
Novelty: High
Scholarly Presentation: High
Is the paper likely to interest a substantial number of physical chemists, not just specialists working in the authors' area of research?: Yes

Reviewer: 2
Recommendation: This paper may be publishable, but major revision is needed; I would like to be invited to review any future revision.

Comments:
The paper of Lokamani et al. presents a computational study of the electronic coupling of a molecule thiol groups to gold. The results are compared to MCBJ measurements. The main novelty of the study is the theoretical approach used, relies on sampling and weighing possible configurations of the molecule. I think this approach is a reasonable alternative to analyzing geometries from molecular dynamics trajectories. The approach described here is novel and could be a significant contribution. I think the paper could be published, however some changes are needed first.
The procedure used for generating and evaluating trajectories should be described in more detail. If it, after all, the main contribution of the paper but its description is displaced instead to the supplemental section. The authors should specify here the advantages of their proposed approach over MD trajectories.
Answer:
We have restructured our manuscript and moved the experimental- and theoretical-methods sections in front and highlight the advantages of our dynamical simulation approach. The technical details and various steps of our approach have been described in the SI.

It is sometimes hard to follow the discussion of the trends in figure 2 without seeing the structure of the scattering region. Can the authors add snapshots in the supplementary section? Why are configurations where S is adsorbed on the planar part of the pyramid relevant at certain distances (figure 4e) but neither before nor after?
Answer:
We thank the reviewer for making this suggestion and have added representative configuration in the supplements for various anchoring positions and certain tip-tip separations (see SI Section S12).

In reality, configurations where S is adsorbed on the planar part of the pyramid are present for all the tip-tip separations in the regions I and II (see Figure 3(a) gray curve). In Figure 4 (now 5), we only show 50 configurations with the highest random walk weights, in order to avoid cluttering. Only in regions III and IV we have predominately tip-edge and tip-tip configurations (see Figure 3(a) red and brown curves).

Why does Gamma increase when the junction is breaking? I would expect it do decrease as the contact is broken.
Answer:
We assume, that the reviewer is referring to the region IV (see Figure 2), where the molecule is still anchored predominately between the tip apexes. We refer to the new section on representative configurations in the supplements (SI Section S12). In this region, the molecule can form the optimal Au-S-C anchoring angle, as described in the manuscript. This in turn leads to an enhancement in the electronic coupling between the metallic states and the dominant transport channel of the molecule. Only beyond the region IV, the junction is broken, where the molecule bonds only to one of the electrodes. For such configurations, $\Gamma_{max/min}$ cannot be determined theoretically using the EGF-method.

Supplemental figure 10 shows some examples of T(E) and their fits. Many details of the fit are missing. What range are they fitted over? Sub-figure 1 (#1) is a terrible fit that should be rejected. Is there any goodness-of-fit criteria applied or are all fits (such as #1) included regardless of their quality? What do the data look like on a logarithmic scale?
Answer:
We have added a new section on curve-fitting for measurements (SI Section S1) and have updated the

curve fitting (see SI Section S8) with more details. We do acknowledge the fact that some of the fits to the transmission function calculated using EGF are not up to mark. But we do not exclude these relevant configurations with high random walk weights. A more accurate comparison would require to extend the theoretical model beyond the single level model and include experimental measurements which do not correspond to the single-level-model. We intend to current study in future works.

Additional Questions:
Urgency: High
Significance: High
Novelty: High
Scholarly Presentation: Moderate
Is the paper likely to interest a substantial number of physical chemists, not just specialists working in the authors' area of research?: Yes

Reviewer: 3
Recommendation: This paper may be publishable, but major revision is needed; I would like to be invited to review any future revision.
Comments:
The manuscript of M. Lokamani et al. describes a theoretical model of the electronic molecule-electrode coupling evolution along the stretch of a molecular junction based on thiolate PEEB compound between gold electrodes. It explores the contribution of three geometrical descriptors: the anchoring sites, the curvature of the molecular backbone and the thiol-electrode binding angle. The theoretical results are related to some examples of the evolution of the parameter Gamma obtained from fitting experimental current-voltage (IV) curves recorded during the stretch of a Salen compound / gold molecular junction to the single level model (SLM). In my opinion, this work suggest interesting contributions to the electronic coupling in a molecular junction in an exhaustive and comprehensive way and I think the manuscript should be published. However, there are some points that the authors must address before publication:
Regarding the experimental section:

1) The experimental section in the manuscript is very brief. Relevant information is missing. For instance the authors mention that 700 stretching cycles were studied, but give not statistics in how many of them reproduce the behavior of interest during the stretching: an initial decrease of Gamma followed by a somehow flat region with individual peaks and finally a new Gamma increase. Was this the main observed behavior or a marginal one? Which was the main observed behavior in the latter case? (only three stretching examples are given in the manuscript). Although the fact the behavior could be not frequent would not mean that it is not interesting, this information should be given in the manuscript.
Answer:
We observed partial signatures of the stretch evolution of Γ for Co-Salen in 5 measurements. We observe the complete evolution with the rising and falling trend, and an intermediate flattened region for only one measurement. We have included this information in the revised manuscript.
(Main manuscript page 6 first paragraph)

2) Additionally, how fast were the IV curves recorded in the experiment? The system was given time to relax after each stop before recording the IV curves? Were both IV curves recorded at a given point identical?
Answer:
The I-V measurements were recorded following a butterfly sweep. We wait at least 90 s between the I-V measurements following a butterfly sweep and until the standard deviation of the measured signal is lower than a threshold. The opening speed of the tips is of the order of $2 \times 10{-}12$ m/s. Both the IV curves were not identical and we averaged the Gamma values obtained from the 2 curves per position. We have included the details on the measurements in the manuscript and have added another reference for technical details on previous experiments.
(Main manuscript page 5 lower half)

3) The authors should give examples of the 'detected molecular signatures' that they used as evidence that a molecular junction had formed, as well as the examples of experimental IV curves and their fits at least in the supporting information (SI) document (fits to the theoretical T(E) curve are indeed included in this document). In particular, which expression was fitted to the experimental IV curves? Only one Gamma is given per position. Does this mean that only symmetric fits were considered, or this Gamma is the average of Gamma_min and Gamma_max?
Answer:
We have included a new section on curve-fitting of the measured IV-curves in the supplements. In this section, we discuss the details of our curve-fitting procedure for the measured IV-curves. We have also

added a reference to our earlier work, where we discuss the detection of the molecular signal in detail. We consider asymmetric fits and evaluate Gamma which is the average of Gamma_min and Gamma_max.
(Main manuscript page 5 lower half, SI Section S1)
(Reference 16)

4) An experimental Gamma decrease reflects mainly a conductance decrease. Can the decreasing direct tunneling contribution account for the experimental Gamma decrease during the initial part of the stretching?
Answer:
We assume that the reviewer refers to direct tunneling between the Au electrodes. In fact this would be a possibility, since for the geometry studied here, the current path through the molecule in the initial situation is longer than the spacing between the Au electrodes. We would, however, expect that direct tunnelling through the solvent does not lead to IV-curves which match the single level model with very high goodness of fit (see, for example, K. Luka Guth et al., *Role of Solvents in the Electronic Transport Properties of Single-Molecule Junctions*, Beilstein J. Nanotechnol. 7, 1055 (2016)). It is therefore more likely that we see transport through a single molecule even at small distances than that we observe direct tunneling. This assumption is further supported by the fact that also in later stages of the stretching we see transport through the molecule.

5) Why was this particular Salen compound used in this work when the theoretical calculations were done for a simpler PEEB compound? Were experiments done with PEEB as well? If so, were Gamma showing the same evolution? These points should be clarified in the manuscript.
Answer 5:
We thank the reviewer for pointing out this difference. At tip-tip separations of the order of the dimension of the PEEB molecules, tunneling currents between the tip electrodes dominate over the molecular signature. As a result, stable measurements for determining the stretch evolution of PEEB were not possible. We have mentioned the reason for the comparison of different molecules in the experiments and theoretical analysis in the revised manuscript.
(Main manuscript page 6 lower half)

6) The magnitude d_0 mentioned at the end of section 1 of the SI document is not defined. What does it mean?
Answer 6:
We thank the reviewer for pointing out this missing piece of information. d_0 denotes the position of the motor used for pushing the pin in the MCBJ setup.
(SI Section S1 Data evaluation)

Regarding the model:
7) What magnitude is exactly quantified and represented in Figure 2(a) as AP? Is it the probability weight of each configuration? Which are the units? Please clarify in the manuscript.
Answer 7:
We have reformulated the sentence in the manuscript. AP quantifies the fraction of configurations with various combinations of anchoring positions on a scale between 0 and 1.
(Main manuscript page 8 lower half)

8) In the theoretical section, what does it mean 'thermodynamical weights according to individual energies and involved energy landscapes'? Please clarify in the manuscript.

Answer 8:
We have reformulated the sentence in the manuscript.

9) In the description of Figure 13, the authors describe a 'strong correlation' of eplison_0 with the left and right anchoring angles but that is not observed in Figure 13. First, the intersection of these magnitudes is blue, so there is a negative correlation or anticorrelation between them. Then, the colors is quite light pointing to a light or mild anticorrelation. Similarly, the intersection between the Gammas and mC presents a very light color but the text describes a strong correlation between them. Other positive and negative correlations are stronger in the figure but not highlighted. Also in this figure, why the correlation of the right and left angles with other magnitudes are different?

10) In particular in the same Figure 13, S_tip-tip has a negative correlation with AP_tip which puzzles me: as I understand it, for high S_tip-tip the tip-tip configuration is dominant, shouldn't it be a strong positive correlation between these magnitudes? Which is the definition of correlation used in this work? Please clarify.
Answer 9/10:
We thank the author for pointing out these discrepancies. We realized a mismatch in our labels which we have corrected.
(SI Section S10).

11) Other comments:
- In my opinion the manuscript is not well structured and results difficult to read. The experimental and theoretical sections are at the end of the document, but the results section cannot read or understood without reading them first as not summary or explanation of what has been done in presented in this section. No even the magnitudes as Gamma_min and Gamma_max for instance are defined. Also part of the essential information to understand the results is presented in the SI document. I understand the need to be brief in a Letter, but I consider that the text should be consistent and easy to read without continuous going backs and forwards.
Answer:
We initially followed the order of the section recommended in the author guide for the journal. We have restructured the manuscript and moved the theoretical and experimental section in front in order to improve the readability of the manuscript.

- In particular, the acronyms are not properly introduced: SLM is only introduced in Figure 1 caption, SCC-DFTB in the conclusions or PEEB is introduced after being used several times.
Answer:
We have corrected the order of acronyms in the manuscript.

- The name of the first author is missing.
Answer:
We have corrected the name.

- References to figures are sometimes confusing as those of the SI document are not numbered as Figure SX.
Answer:
We have corrected the numbering.

- In section 7 of the SI document (Figure 9): two names are used for the same magnitude: n_(SL-C) and n_(L-SC). I guess SC accounts for sulfur-carbon, but please define.

Answer:
We have added a description for [L|R]_SC.

Additional Questions:
Urgency: Moderate
Significance: High
Novelty: High
Scholarly Presentation: Moderate
Is the paper likely to interest a substantial number of physical chemists, not just specialists working in the authors' area of research?: Yes

jz-2023-00370f.R2

Name: Peer Review Information for "Stretch Evolution of Electronic Coupling of the Thiophenyl Anchoring Group with Gold in Mechanically Controllable Break Junctions"

Second Round of Reviewer Comments

Reviewer: 2

Comments to the Author

I think the paper of Lokamani et al. is improved in this revised version due to the changes suggested by all reviewers. In particular, the description of the method (general approach and also details of the implementation) is better described now. I believe the high-throughput simulation described here provides insight on the relation of Gamma and microscopic structure and will be a valuable contribution to the molecular electronics community. I recommend the paper for publication, but suggest the authors consider the following (optional) further recommendations

The authors updated the T(E) curve-fitting section S8 to address my comment. They mention that T(E) cannot be fitted "for a subset" of configurations and they keep the fitted parameters but they do not say how big this subset is. The authors should add the fraction of configurations that cannot be fitted and comment on whether excluding this subset would change the main conclusions in any region. I suspect not but it would cost nothing to add this information.

In figure 5 the circles indicate the most favorable anchoring positions in pairs. But in regions I and IV, the configurations are predominantly symmetric (edge-edge and tip-tip), in regions II and III they are asymmetric (tip-edge). Could this be somehow included in figure 5 by choosing different colors for symmetric and asymmetric configurations? For example red for edge-edge, tip-tip, blue for tip-edge. Related to this, would this asymmetry be seen if the cross-correlation plot in figure S14 were repeated for regions I to IV independently? I think this could add extra information about the microscopic structures to the figures.

Author's Response to Peer Review Comments:

Dear Editor,

thank you for your response and decision to accept the manuscript.

I have uploaded the necessary files (Coverart and TOCGraphic) and also uploaded the supplementary with a minor change in Figure S4(a) on page S7. The label WRM Emin has been changed to WBM Emin according to the caption.

Feel free to contact me if you have any further questions or queries.

Yours Sincerely

Lokamani
